# Cardiometabolic risk factors for COVID-19 susceptibility and severity: A Mendelian randomization analysis

**DOI:** 10.1371/journal.pmed.1003553

**Published:** 2021-03-04

**Authors:** Aaron Leong, Joanne B. Cole, Laura N. Brenner, James B. Meigs, Jose C. Florez, Josep M. Mercader

**Affiliations:** 1 Department of Medicine, Harvard Medical School, Boston, Massachusetts, United States of America; 2 Division of General Internal Medicine, Massachusetts General Hospital, Boston, Massachusetts, United States of America; 3 Programs in Metabolism and Medical and Population Genetics, Broad Institute of MIT and Harvard, Cambridge, Massachusetts, United States of America; 4 Diabetes Unit and Center for Genomic Medicine, Massachusetts General Hospital, Boston, Massachusetts, United States of America; 5 Division of Endocrinology and Center for Basic and Translational Obesity Research, Boston Children’s Hospital, Boston, Massachusetts, United States of America; 6 Division on Pulmonary and Critical Care, Massachusetts General Hospital, Boston Massachusetts, United States of America; Université Laval, CANADA

## Abstract

**Background:**

Epidemiological studies report associations of diverse cardiometabolic conditions including obesity with COVID-19 illness, but causality has not been established. We sought to evaluate the associations of 17 cardiometabolic traits with COVID-19 susceptibility and severity using 2-sample Mendelian randomization (MR) analyses.

**Methods and findings:**

We selected genetic variants associated with each exposure, including body mass index (BMI), at *p <* 5 × 10^−8^ from genome-wide association studies (GWASs). We then calculated inverse-variance-weighted averages of variant-specific estimates using summary statistics for susceptibility and severity from the COVID-19 Host Genetics Initiative GWAS meta-analyses of population-based cohorts and hospital registries comprising individuals with self-reported or genetically inferred European ancestry. Susceptibility was defined as testing positive for COVID-19 and severity was defined as hospitalization with COVID-19 versus population controls (anyone not a case in contributing cohorts). We repeated the analysis for BMI with effect estimates from the UK Biobank and performed pairwise multivariable MR to estimate the direct effects and indirect effects of BMI through obesity-related cardiometabolic diseases. Using *p <* 0.05/34 tests = 0.0015 to declare statistical significance, we found a nonsignificant association of genetically higher BMI with testing positive for COVID-19 (14,134 COVID-19 cases/1,284,876 controls, *p =* 0.002; UK Biobank: odds ratio 1.06 [95% CI 1.02, 1.10] per kg/m^2^; *p =* 0.004]) and a statistically significant association with higher risk of COVID-19 hospitalization (6,406 hospitalized COVID-19 cases/902,088 controls, *p =* 4.3 × 10^−5^; UK Biobank: odds ratio 1.14 [95% CI 1.07, 1.21] per kg/m^2^, *p =* 2.1 × 10^−5^). The implied direct effect of BMI was abolished upon conditioning on the effect on type 2 diabetes, coronary artery disease, stroke, and chronic kidney disease. No other cardiometabolic exposures tested were associated with a higher risk of poorer COVID-19 outcomes. Small study samples and weak genetic instruments could have limited the detection of modest associations, and pleiotropy may have biased effect estimates away from the null.

**Conclusions:**

In this study, we found genetic evidence to support higher BMI as a causal risk factor for COVID-19 susceptibility and severity. These results raise the possibility that obesity could amplify COVID-19 disease burden independently or through its cardiometabolic consequences and suggest that targeting obesity may be a strategy to reduce the risk of severe COVID-19 outcomes.

## Abstract 2

Aaron Leong and co-workers investigate causal risk factors for COVID-10 illness and severity.

## Author summary

**Why was this study done?:**

- Diverse cardiometabolic risk factors have been described in the literature to be associated with COVID-19 illness, but causality has not been established.
- Preventive strategies targeting cardiometabolic risk factors that are both causal and modifiable may reduce the risk of COVID-19 illness, whereas interventions targeting risk factors that are only correlated with the outcome may not.

**What did the researchers do and find?:**

- We used 2-sample Mendelian randomization analyses to test whether 17 cardiometabolic diseases and traits had a causal relationship with risk of COVID-19 illness.
- We found that higher body mass index was the only cardiometabolic risk factor among those we studied that was associated with a higher risk of hospitalization for COVID-19 compared to the general population.
- Obesity-related cardiometabolic diseases—type 2 diabetes, chronic kidney disease, stroke, and coronary heart disease—may be mediators of the relationship between body mass index and higher risk of hospitalization for COVID-19.

**What do these findings mean?:**

- Our results suggest that people with a higher body mass index have a higher risk for hospitalization for COVID-19.
- If other cardiometabolic risk factors have causal associations with COVID-19 illness, their effects are likely modest.
- We provide genetic evidence supporting body mass index as a causal risk factor for COVID-19 severity, raising the possibility that obesity could have amplified the COVID-19 pandemic, directly or through obesity-related cardiometabolic diseases.

## Introduction

There is high heterogeneity in both susceptibility to and severity of SARS-CoV-2 infection, with clinical severity [1,2] ranging from asymptomatic infection to life-threatening respiratory failure and death [3]. Epidemiological studies using both retrospective and prospective cohorts of different sizes and from multiple countries have reported evidence that underlying cardiometabolic conditions [4–29] may be associated with an increased risk of severe COVID-19 illness (i.e., hospitalization, intubation, mechanical ventilation, or death [30]). Coronary artery disease [4,25,29], chronic kidney disease [5–9,28], obesity [10–14], and type 2 diabetes [6,15–18] have strong and consistent evidence for association with COVID-19 severity [30]. There is less compelling evidence for cerebrovascular disease [19–26] (i.e., stroke) and hypertension [4,24–27] leading to severe manifestations of COVID-19. Additional evidence suggests that cardiometabolic traits may be associated with disease susceptibility [31]; however, without universal testing, this correlation is difficult to prove.

While early reports are crucial to inform clinical decision making and public health policy during a pandemic of a new pathogen, correlative observational data can be plagued by residual confounding. Thus, there remain inherent challenges in inferring causal impact from these epidemiological studies. Mendelian randomization (MR) is an analytic approach that uses human genetic variation known to influence modifiable exposures to examine their causal effect on disease [32]. MR is especially useful for disentangling causal pathways of phenotypically clustered risk factors that are difficult to randomize or prone to measurement error. By identifying causal relationships between cardiometabolic risk factors and COVID-19 susceptibility or severity, we may be able to mitigate their impact on disease risk and avoid spurious conclusions that lead to misinformation or incite unnecessary anxiety.

We hypothesize that only some cardiometabolic conditions have a causal relationship with COVID-19 illness or its disease course. Thus, we sought to evaluate the associations of 17 cardiometabolic exposures with COVID-19 susceptibility and severity using 2-sample MR analyses. MR effects were estimated from genome-wide association study (GWAS) summary statistics of these cardiometabolic diseases and related traits and COVID-19-related outcomes from the COVID-19 Host Genetics Initiative (https://www.covid19hg.org/) [33].

## Methods

We selected 17 cardiometabolic traits and diseases that cluster clinically with metabolic syndrome, obesity, diabetes, and their complications: type 1 diabetes [34], type 2 diabetes [35], hemoglobin A1c [36], fasting glucose adjusted for body mass index (BMI) [36], fasting insulin adjusted for BMI [36], BMI [37], waist–hip ratio adjusted for BMI [38], low-density lipoprotein cholesterol [39], high-density lipoprotein cholesterol [39], triglycerides [39], systolic blood pressure [40], diastolic blood pressure [40], creatinine-based estimated glomerular filtration rate (eGFR) [41], chronic kidney disease [41], coronary artery disease [42], any stroke [43], and C-reactive protein (CRP) [44], a nonspecific biomarker of inflammation that can be elevated in people with high cardiometabolic risk. As our study was conducted to narrowly test an a priori hypothesis, we did not have a prespecified analysis plan. This study is reported according to the Strengthening the REporting of Genetic Association Studies (STREGA; S1 STREGA Checklist).

### Candidate instrument selection for cardiometabolic diseases and traits

We extracted association summary statistics from published large-scale GWAS meta-analyses to generate sets of genetic instruments for each of these exposures. We used genetic variants associated with these exposures at genome-wide significance (*p <* 5 × 10^−8^) and excluded those that were not represented in the COVID-19 outcome GWAS datasets. Using the LD_clumping function, we pruned the list of candidate instruments for linkage disequilibrium (LD; *r*^2^ > 0.01) and discarded variants that were within 1-Mb distance from other candidate instruments with a stronger association. Analyses were performed using the R package TwoSampleMR v.4.0 [45,46].

### COVID-19 Host Genetics Initiative GWAS meta-analysis for susceptibility and severity

The COVID-19 Host Genetics Initiative is an international genetics collaboration that aims to uncover the genetic determinants of outcomes related to COVID-19 susceptibility and severity [33]. To accomplish this, investigators from around the world assembled individual-level clinical and genetic data and performed individual GWASs. All cohorts imputed genotypes to Haplotype Reference Consortium [47], 1000 Genomes [48] or TOPMed [49] reference panels. Each contributing cohort defined ancestry by self-report or genetic data and performed single-variant association testing adjusting for age, age^2^, sex, age × sex, genetic ancestry principal components, and other study-specific covariates. Summary statistics were shared via a cloud-based computing platform for centralized meta-analysis. An allele frequency filter of 0.0001 and an INFO filter of 0.6 was applied to each study prior to meta-analysis with inverse-variance weighting (IVW). Summary statistics from the fourth round of GWAS meta-analysis, shared publicly on October 20, 2020, were used to test the 17 sets of genetic instruments against COVID-19 outcomes assembled by the COVID-19 Host Genetics Initiative. A total of 22 cohorts had contributed to the GWASs of COVID-19 outcomes used in our primary analyses. Cohorts contributing the largest number of cases (>1,000) included the UK Biobank, deCODE, FinnGen, Million Veteran Program, Ancestry, COVID19-Host(a)ge, and GenOMICC. Participants were mostly healthy volunteers or people seeking medical care within a healthcare system. To our knowledge, none of the participants were recruited via random sampling.

For our 2 primary analyses, we restricted analysis to individuals of European ancestry only, and selected the COVID-19 outcomes with the largest number of cases. Susceptibility was defined as testing positive for COVID-19 by reverse transcription quantitative polymerase chain reaction (RT-qPCR), serological testing, or clinician diagnosis by chart review or ICD coding (*N =* 14,134) versus population controls (*N =* 1,284,876), which included any person who was not a case (i.e., people who tested negative, were never tested, or had an unknown testing status. Severity was defined as hospitalization of patients with COVID-19 by RT-qPCR, serological testing, or clinician diagnosis (*N =* 6,406) versus population controls (*N =* 902,088). As controls were not selected based on testing results, specific characteristics, or testing status, they were likely to be representative of the general population.

To determine whether statistically significant results from the primary analyses were consistent across different definitions for COVID-19 susceptibility, severity, and control groups, we performed secondary MR analyses of the 5 remaining outcomes that were made available by the COVID-19 Host Genetics Initiative without restriction by ancestry. For susceptibility, these outcomes were (1) COVID-19 positive by RT-qPCR, serological testing, or clinician diagnosis (*N =* 24,057) versus COVID-19 negative by RT-qPCR, serological testing, or self-report (*N =* 218,062) and (2) predicted COVID-19 based on symptoms or COVID-19 positive by self-report (*N =* 3,204) versus no predicted COVID-19 based on symptoms or no COVID-19 by self-report (*N =* 35,728) using a model developed by Menni et al. [50]. For severity, these outcomes were (1) critical respiratory illness, defined by death, intubation, continuous positive airway pressure (CPAP), bilevel positive airway pressure (BiPAP), continuous negative external pressure (CNP), or very high flow positive end expiratory pressure oxygen in patients with COVID-19 by RT-qPCR, serological testing, or clinician diagnosis (*N =* 4,933) versus population controls (*N =* 1,398,672); (2) critical respiratory illness (*N =* 269) versus no hospitalization for COVID-19 within 21 days of testing positive for COVID-19 (*N =* 688); and (3) hospitalization (*N =* 2,430) versus no hospitalization (*N =* 8,478) among people with COVID-19.

### MR analysis of COVID-19 susceptibility and severity

To estimate the association of each exposure with each outcome, we performed 2-sample MR analyses using the random-effects IVW method, whereby genetic variant–outcome coefficients were modeled as a function of genetic variant–exposure coefficients weighted by the inverse of the squared genetic variant–outcome standard errors [51]. The use of random effects provides a concise estimation and considers potential heterogeneity among estimates from individual variants [52]. We used *p <* 0.05/17 exposures/2 outcomes = 0.0015 to declare statistical significance, with the understanding that this threshold may be conservative as exposures are clinically correlated. We reported MR effect estimates as odds ratios for the outcome per log-odds of binary exposures or unit change of continuous exposures. For BMI, we repeated the analysis using untransformed variables from UK Biobank (http://www.nealelab.is/uk-biobank) to report MR effect estimates per unit change of raw BMI.

### Accounting for pleiotropy

An assumption of MR is that instruments do not influence the outcome independently of the risk factor of interest, i.e., non-mediated pleiotropy. We tested this assumption in a series of sensitivity analyses. We used the weighted median estimator (WME) [53], which requires ≥50% of the contribution to the MR estimate to be from valid instruments; if so, its MR estimate is stable. We then used the MR-Egger regression [54], whereby a linear regression of variant–outcome on variant–exposure coefficients was performed without constraining the intercept to the origin. The slope of the regression line provides the corrected MR estimate even when none of the instruments are valid [54]. Next, we used the mode-based estimate, which is consistent when the largest number of similar single-variant MR estimates are derived from valid instruments even when the majority are invalid [55]. If all MR models produced similar MR estimates despite making different assumptions on the validity of instruments, we would be more confident of the robustness of our results [56]. In other sensitivity analyses, we applied MR pleiotropy residual sum and outlier (MR-PRESSO) [57] and leave-one-out analysis to determine whether outliers may be biasing the overall MR estimate.

To estimate direct and indirect effects of BMI via obesity-related cardiometabolic diseases (coronary artery disease, stroke, chronic kidney disease, and type 2 diabetes), we performed pairwise multivariable MR wherein we conditioned upon the effects of these exposures with BMI one at a time. Using GWASs with full summary statistics, we extracted the summary statistics of all the variants that had reached genome-wide significance in at least 1 of these exposures. As adding variants that are not associated with BMI for the purpose of jointly predicting multiple exposures in multivariable MR may weaken the instrument, we calculated the Q statistic to test for weak instruments using methods by Sanderson et al. [58], and confirmed that the instruments had sufficient strength to predict each trait. We aligned the variants to the reference allele, excluding the palindromic variants with alternate allele frequency between 0.4 and 0.6 using the harmonize_data function. Using the function mv_multiple, we fitted these 4 cardiometabolic diseases with BMI one at a time. We considered results significant when *p =* 0.05/4/2 = 0.006 as we tested 4 pairs of exposures for 2 outcomes.

## Results

### Selection of genetic instruments for exposures

We obtained genetic instruments for the 17 exposures for MR analyses after excluding variants that were in LD (*r*^2^ > 0.01) and in proximity (1 Mb) to other candidate instruments with stronger *p*-values. Genetic instruments explained 0.2% to 5.3% of the variance or liability of each exposure (Table 1). Contributing studies included in these exposure GWAS meta-analyses were predominantly of individuals of European ancestry.

**Table 1:** Candidate genetic instruments of cardiometabolic diseases and traits.

Exposure	Adjusted for BMI	PMID or reference	Sample size, *N*	Ancestry of participants	Candidate genetic instruments, *N*	Genetic instruments used in analysis, *N*	Estimated variance explained (%)
Type 1 diabetes	No	25751624	6,808 cases/12,835 controls	European	75	50	3.2
Type 2 diabetes	No	30297969	898,130 (9% cases)	European	243	226	3.1
Hemoglobin A1c	No	Chen et al. [36]	Up to 281,416	70% European	216	105	2.2
Fasting glucose	Yes	Chen et al. [36]	Up to 281,416	70% European	179	91	1.6
Fasting insulin	Yes	Chen et al. [36]	Up to 281,416	70% European	96	61	1.0
BMI	No	25673413	Up to 339,224	Mostly European	75	72	1.7
Waist–hip ratio	Yes	25673412	Up to 224,459	Mostly European	53	43	0.8
C-reactive protein	No	31900758	Up to 418,642	European	439	437	5.3
Low-density lipoprotein	No	24097068	Up to 188,577	European	65	63	1.9
High-density lipoprotein	No	24097068	Up to 188,577	European	54	53	1.8
Triglycerides	No	24097068	Up to 188,577	European	39	38	1.3
Systolic blood pressure	No	30224653	>1,000,000	European	185	181	1.5
Diastolic blood pressure	No	30224653	>1,000,000	European	190	183	1.5
Creatinine-based eGFR	No	31152163	>1,000,000	Mostly European	547	280	3.3
Chronic kidney disease	No	31152163	64,164 cases/561,055 controls	Mostly European	23	21	0.6
Coronary artery disease	No	28714975	10,801 cases/137,914 controls	Mostly European	50	50	0.9
Any stroke	No	29531354	67,162 cases/454,450 controls	Mostly European	23	16	0.22

The number of candidate genetic instruments refers to the number of variants that were associated with the exposures at genome-wide significance (*p <* 5 × 10^−8^) in GWASs. The number of genetic instruments used in the analysis refers to the number of variants that were used in the Mendelian randomization analysis after excluding those that were not represented in the COVID-19 GWAS, or were in linkage disequilibrium or within 1-Mb distance from other candidate instruments with a stronger association. We used the sex-combined summary statistics for BMI and waist–hip ratio adjusted for BMI. All other exposures were adjusted for sex. Where indicated, we used summary statistics adjusted for BMI. We elected to use effect estimates from European-specific GWASs or from multi-ancestry GWASs where the bulk of the data were provided by participants of European descent. Sample sizes were the maximum number indicated in the published source. Estimated variance explained by genetic instruments was the sum of estimated variance explained by each variant calculated from reported *p*-values, sample sizes, and proportion of cases and controls using the TwoSampleMR R functions get_r_from_lor() and get_r_from_pn().

BMI, body mass index; eGFR, estimated glomerular filtration rate; GWAS, genome-wide association study; PMID, PubMed identifier.

### MR effect of each cardiometabolic exposure on COVID-19 susceptibility and severity

**Fig 1:**
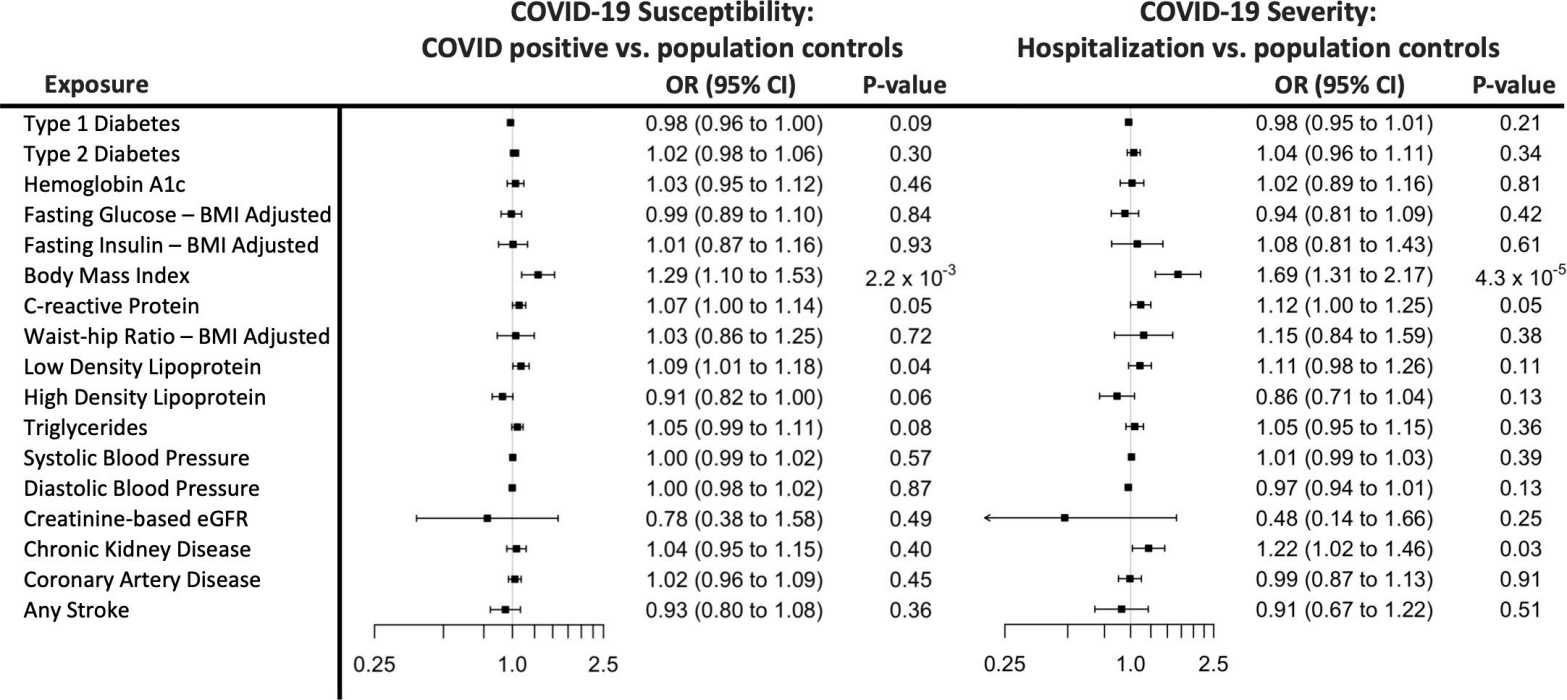
Forest plot Mendelian randomization (MR) effect estimates and 95% confidence intervals for each exposure and the 2 main outcomes analyzed. MR estimates are reported as odds ratios (ORs) per unit of the exposure: hemoglobin A1c, percent unit; fasting glucose, mg/dl; fasting insulin, natural log; body mass index (BMI), inverse normally transformed residuals; waist–hip-ratio, inverse normally transformed residuals; C-reactive protein, rank-based inverse normally transformed; low-density lipoprotein, standardized; high-density lipoprotein, standardized; triglycerides, standardized; systolic and diastolic blood pressure, mm Hg; estimated glomerular filtration rate (eGFR), ml/min/1.73 m^2^; type 1 diabetes, type 2 diabetes, coronary artery disease, chronic kidney disease, and any stroke, log-odds.

Of the 17 cardiometabolic exposures, only BMI was found to be associated with COVID-19 severity after accounting for multiple testing (*p <* 0.0015; Fig 1). We found a nonsignificant association of genetically higher BMI with a higher risk of testing positive for COVID-19 after correcting for multiple testing (*p =* 0.002), and a significant association with a higher risk of COVID-19 hospitalization (*p =* 4.3 × 10^−5^), compared to population controls using random-effects IVW (Fig 1).

**Fig 2:**
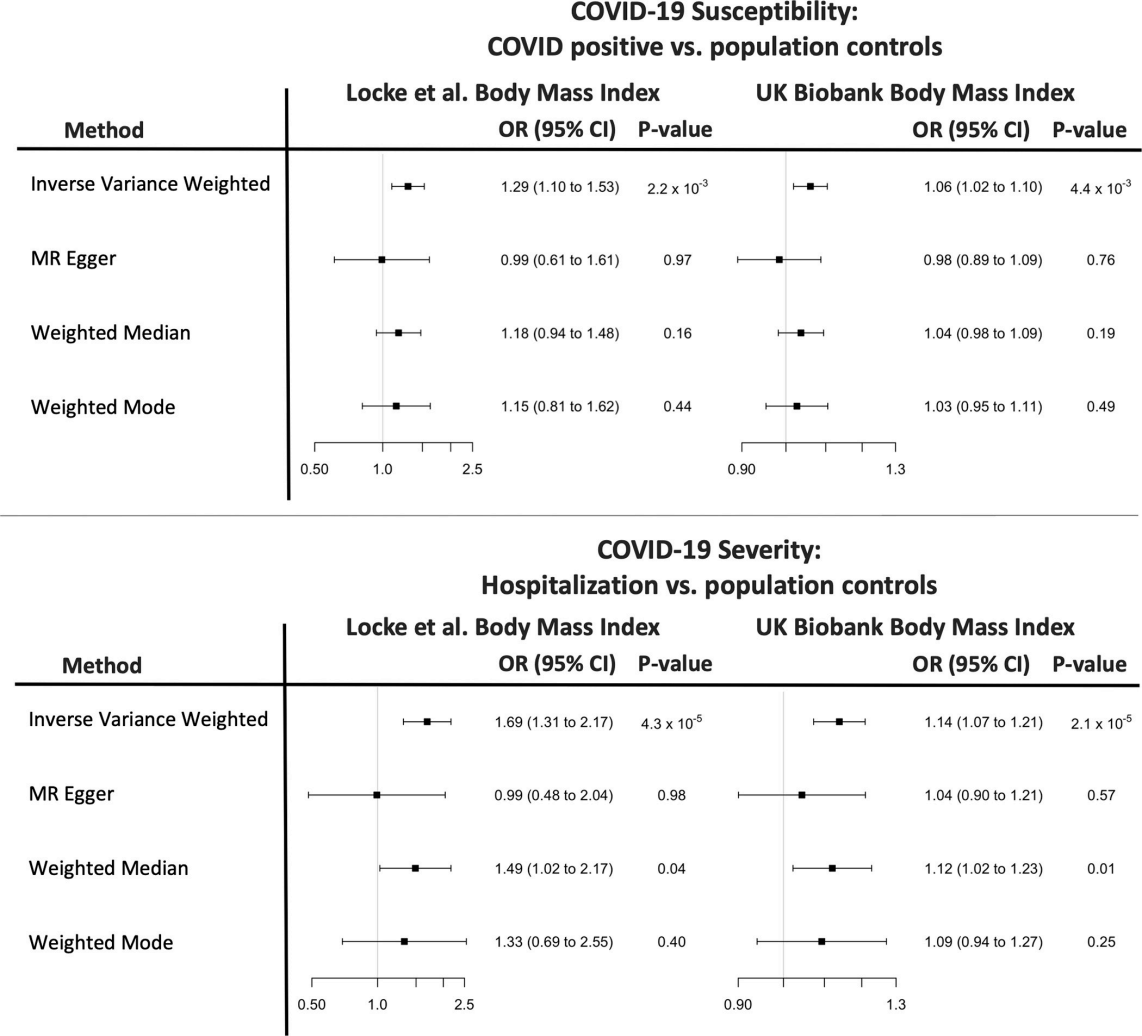
Sensitivity analyses using other Mendelian randomization (MR) methods and results using UK Biobank effect estimates. MR estimates are reported as odds ratios (ORs) per unit increase in body mass index (BMI). Locke et al. [37]: inverse normally transformed residuals; UK Biobank: kg/m^2^.

Out the 1,984 genetic instruments used for the 17 cardiometabolic exposures, 8 had an F-statistic < 10; none of them had been used for BMI (Table A in S1 Tables). Excluding the few variants with F-statistic < 10 and using a LD clumping threshold of *r*^2^ < 0.001 did not materially change the results for the any of the cardiometabolic risk factors (Fig A in S1 Figs). For both outcomes, we identified no heterogeneity of effects (*p =* 0.06; *p =* 0.25) or outlying genetic variants by the leave-one-out analysis or MR-PRESSO (Figs B–G in S1 Figs). To obtain interpretable effect estimates, we repeated the analysis using beta estimates of raw BMI from UK Biobank [59] and found consistent results: an odds ratio of 1.06 per kg/m^2^ increase in BMI (95% CI 1.02, 1.10; *p =* 0.004) for testing positive with COVID-19, and an odds ratio of 1.14 per kg/m^2^ increase in BMI (95% CI 1.07, 1.21; *p =* 2.1 × 10^−5^) for COVID-19 hospitalization. Point estimates from the MR-Egger, WME, and weighted mode analyses, were in the same direction as those from the IVW analysis (Fig 2). The MR-Egger intercept *p* was 0.25 and 0.13 for susceptibility and severity, respectively, indicating the absence of directional pleiotropy.

### MR of 5 other COVID-19 outcomes in secondary analyses

Genetically higher BMI was associated with a higher risk of critical respiratory illness versus population controls (*p =* 7.7 × 10^−4^) and of testing positive for COVID-19 versus testing negative for COVID-19 (*p =* 0.03). We found a nonsignificant association of CRP with hospitalization versus no hospitalization among people with COVID-19 (*p =* 0.002). The associations of other cardiometabolic exposures with these 5 COVID-19 outcomes were null (Figs H–Q in S1 Figs). As studies have reported an association between critical illness in COVID-19 and hypercoagulability [60], we tested the association of venous thromboembolism with all 7 COVID-19 outcomes using 41 genetic instruments extracted from a GWAS of venous thromboembolism in people of mostly European ancestry [61]. All associations were null (Fig R in S1 Figs).

### Multivariable MR analysis with BMI

The implied direct effects of BMI on the 2 COVID-19 outcomes were abolished upon conditioning on the genetic effects of each of the 4 obesity-related cardiometabolic diseases—coronary artery disease, stroke, chronic kidney disease, and type 2 diabetes (BMI, *p* > 0.0125; Table 2). In the multivariable model with both BMI and chronic kidney disease, we observed an association between the risk of chronic kidney disease and the risk of COVID-19 hospitalization (odds ratio 1.25 [95% CI 1.08, 1.42] per log-odds, *p =* 0.01), though this association was not statistically significant after accounting for multiple testing.

**Table 2:** Direct effect of BMI and other obesity-related cardiometabolic diseases on COVID-19 susceptibility (testing positive) and severity (hospitalization) versus population controls in pairwise multivariate Mendelian randomization.

Model	Exposure	Outcome	
		Susceptibility OR (95% CI), *p-*value	Severity OR (95% CI), *p-*value
BMI + T2D	BMI	1.00 (0.97, 1.03), 0.92	1.01 (0.95, 1.06), 0.82
	T2D	1.02 (0.98, 1.06), 0.29	1.05 (0.97, 1.12), 0.22
BMI + CKD	BMI	1.01 (0.97, 1.04), 0.65	0.99 (0.93, 1.05), 0.66
	CKD	1.06 (0.96, 1.15), 0.28	1.25 (1.08, 1.42), 0.01
BMI + CAD	BMI	1.02 (0.98, 1.05), 0.36	1.03 (0.97, 1.10), 0.30
	CAD	1.04 (0.97, 1.11), 0.31	1.02 (0.90, 1.15), 0.72
BMI + stroke	BMI	1.01 (0.97, 1.04), 0.75	0.98 (0.92, 1.04), 0.57
	Stroke	1.03 (0.88, 1.18), 0.71	1.08 (0.83, 1.33), 0.54

As we used *p =* 0.05/4/2 = 0.006 to declare statistical significance, none of the associations were statistically significant.

BMI, body mass index; CAD, coronary artery disease; CKD, chronic kidney disease; OR, odds ratio; T2D, type 2 diabetes.

To test whether adiposity measures were associated with COVID-19 outcomes, we performed univariable MR analyses for waist–hip ratio, hip circumference, and waist circumference with and without adjustment for BMI. Waist–hip ratio with and without adjustment for BMI was not associated with COVID-19 outcomes. Waist circumference and hip circumference were both nominally associated with testing positive for COVID-19 versus population controls, but not with adjustment for BMI. These results suggest that these adiposity traits were not associated with COVID-19 outcomes independently of BMI (Fig S in S1 Figs).

## Discussion

Cardiometabolic diseases have been identified to be risk factors for COVID-19 illness [30]. Since risk factors may be only correlated, and not causally related, with outcomes of interest, it is paramount to assess causality to inform preventive strategies. Using the 2-sample MR IVW method, we found that genetically higher BMI was the only risk factor for COVID-19 severity among the 17 cardiometabolic diseases and traits tested, whereby the odds of hospitalization with COVID-19 was 14% higher per kg/m^2^ increase in BMI. The association of genetically higher BMI with higher COVID-19 susceptibility did not meet our significance threshold of *p <* 0.0015. While our MR findings were consistent with the multiple epidemiological studies that have reported an association between obesity and COVID-19 illness [10–14], we note that results using other MR methods that are robust to violations of instrumental variable assumptions were null, suggesting that our primary results may be biased by pleiotropy. To disentangle implied direct and indirect effects of BMI, we used a multivariable MR analysis to adjust for the genetic effect on obesity-related cardiometabolic diseases. We showed that the implied direct effect of BMI was eliminated upon conditioning on each of the obesity-related conditions chronic kidney disease, stroke, coronary artery disease, and type 2 diabetes one at a time. These results suggest that the association of BMI may be mediated through its cardiometabolic consequences.

Previous MR studies have reported BMI as a risk factor for COVID-19-related outcomes. Genetically higher BMI was associated with a higher risk of developing sepsis, respiratory failure, and hospitalization with COVID-19 in UK Biobank and the HUNT study [62]. Another MR investigation showed that the association of BMI with COVID-19 outcomes persisted upon conditioning on waist circumference, trunk fat ratio, cardiovascular disease, and type 2 diabetes. It is noteworthy that the type 2 diabetes GWAS used in that analysis was smaller than the one used in our study, which may explain why attenuation of the BMI effect on COVID-19 outcomes in their multivariable MR was not observed [63]. Further, our MR study used a larger and more recently released GWAS of COVID-19 outcomes. In a MR study on the association of physical activity and BMI with COVID-19 outcomes using UK Biobank data, BMI was not found to be associated with COVID-19 (odds ratio 1.37 [95% CI 0.90–2.09] per kg/m^2^, *p =* 0.14) [64], though the confidence interval reported in this study was wider, likely due to its smaller sample size compared to our study.

Apart from BMI, the associations for other cardiometabolic exposures were null. If any of these exposures had a causal role in COVID-19 susceptibility or severity, their effects were likely too small to be detected with our current sample sizes and significance threshold of alpha = 0.0015. Using genetic instruments that explain 1% to 10% of the exposure, our study can detect odds ratios per SD of the exposure ranging from 1.13 to 1.51 (Table B in S1 Tables). Observational correlations of cardiometabolic conditions with COVID-19 outcomes may be partly due to clinical clustering with obesity. It is noteworthy that correlational risk factors can still have clinical utility in identifying at-risk patients even if causality is refuted. However, if preventive efforts only target correlated, but not causal, risk factors, disease risk may not be reduced. As the risks for the cardiometabolic diseases tested vary with age, it is possible that a younger person with a high genetic burden for a disease may not have experienced a disease event that modifies COVID-19 severity. In this scenario, the absence of a significant association does not necessarily imply that having a personal history of the disease has no effect on COVID-19 risk. This is less of a concern for continuous traits such as BMI, in which genetically driven contributions are less dependent on age. Genetic instruments associated with multiple cardiometabolic risk factors may explain why obesity-related cardiometabolic diseases were able to attenuate BMI effects in multivariable MR despite not being associated with COVID-19 outcomes themselves. Future work in well-powered GWASs is needed to examine genetic loci with pleiotropic effects on cardiometabolic risk factors and COVID-19 outcomes to better delineate causal pathways between BMI and COVID-19.

Our study had limitations. The variances explained in the exposures by genetic instruments were modest, though well within the ranges that are typical for complex traits. The use of weak genetic instruments could have limited our ability to detect subtle causal associations and does not exclude the possibility of modest effects. It is also possible that, with larger sample sizes, the association of other cardiometabolic exposures with COVID-19 outcomes could become significant, and confidence intervals would narrow around true estimates. Our analysis did not factor nonlinear exposure–outcome relationships or test for threshold effects at BMI cut-points. Nevertheless, as obesity is commonly defined as a BMI ≥ 30 kg/m^2^, it is reasonable to conclude that the risk associated with obese individuals is higher than that for average-weight individuals. We recognize that collider or selection bias could distort associations [65,66]. As genetic analyses could only be performed on participants who had been nonrandomly selected for genotyping in biobanks, patient registries, hospitals, or population studies, a spurious association between factors that promote entry into genetic studies and COVID-19 hospitalization could occur. Nevertheless, we observe a consistent direction of effect for BMI on COVID-19 across nested samples using various case/control definitions for susceptibility and severity. We also acknowledge that bias towards the null could occur if some people with underlying medical conditions were more likely than the general population to make concerted efforts to lower their own personal risk of viral exposure in response to public health messaging, were more likely to be tested for COVID-19 and receive a negative test result, or were more likely to gain entry into genetic studies. The causal estimates by MR-Egger regression were not as compelling, suggesting that horizontal pleiotropy or other confounding factors could have biased the estimates. Yet, MR-Egger is a less efficient estimator than the other methods [53] and is generally considered as only one of several sensitivity analyses used to evaluate the plausibility of findings. As UK Biobank had contributed to several of the exposure GWASs as well as the outcome GWASs, MR estimates from 2-sample MR could be biased toward observational effect estimates due to participant overlap [67]. Nevertheless, the BMI GWAS by Locke et al. [37] did not include UK Biobank data, and so the association of BMI with COVID-19 outcomes is unlikely to be biased due to overlapping samples. In our primary analyses we chose to use controls that were broadly defined as not being a case. Without universal testing, the control group, albeit representative of the general population, could have been contaminated with people who had contracted COVID-19, particularly those with only mild or no viral symptoms (asymptomatic), which would have biased estimates towards the null. Nonetheless, our results were consistent when using controls that were narrowly defined as people who tested negative for COVID-19.

Our secondary analyses showed that genetically higher BMI was associated with a higher risk of critical respiratory illness versus population controls. Obesity could contribute to the risk of acute respiratory distress syndrome, the main cause of mortality from COVID-19 [68,69]. We did not include critical respiratory illness in our primary analysis because sample sizes were small. Future studies with larger samples are needed to clarify whether the implied causal relationship between BMI and COVID-19 extends to critical respiratory illness. As sex-stratified effect estimates were not provided by the COVID-19 Host Genetics Initiative, we were unable to determine whether BMI effects differed by sex. Contributing cohorts to the COVID-19 Host Genetics Initiative were mostly of European ancestry. Well-powered studies in people of non-European ancestral origins are critically needed as ethnic and racial minorities in the US are disproportionately affected by the pandemic [5,8,24,70–72]. We recognize that the primary social drivers of viral exposure and spread (i.e., crowding within households, wealth and education gaps, working in essential jobs that render social distancing challenging, language barriers, and poor access to healthcare) are likely correlated with, or are themselves, determinants of obesity [73,74]. Future investigations are required to determine whether addressing these upstream social factors mitigates the impact of obesity on COVID-19 outcomes.

Our study provides genetic evidence that supports or refutes causality for a plethora of cardiometabolic conditions, which can inform preventive strategies aimed at modifying risk of COVID-19 illness and deployment of vaccines to high-risk groups.

Among the 17 cardiometabolic exposures tested, only evidence supporting BMI as a causal risk factor for COVID-19 susceptibility and severity was found, consistent with multiple epidemiological studies that have reported an association between obesity and COVID-19 illness. These findings raise the possibility that obesity may have amplified the disease burden of the COVID-19 pandemic either directly or through its metabolic consequences. To the extent that obesity is a modifiable risk factor with a strong environmental component, preventive measures to control the spread of the virus that may promote weight gain (e.g., limitation of access to open spaces for exercise) should be viewed with caution. While any short-term change in weight would be unlikely to influence COVID-19 susceptibility or severity, we highlight the benefits of weight maintenance that extend beyond prevention of obesity-related cardiometabolic conditions to reducing the risk of infection during the pandemic when physical activity may be curtailed. Future research is required to understand the mechanisms through which obesity is associated with a risk of poor health outcomes or mortality, and whether obesity-related conditions are along the causal pathway. Our study has shown how large-scale genotype–phenotype summary data rapidly assembled during a pandemic and made freely accessible to the research community can accelerate research with immediate and direct application to clinical practice and public health messaging.

## Supporting information

S1 STREGA Checklist (DOCX)

S1 Figs (PDF)

S1 Tables (DOCX)

S1 Texts (DOCX)

## Abbreviations

BMI: body mass index
CRP: C-reactive protein
GWAS: genome-wide association study
IVW: inverse-variance weighting
LD: linkage disequilibrium
MR: Mendelian randomization
MR-PRESSO: MR pleiotropy residual sum and outlier
RT-qPCR: reverse transcription quantitative polymerase chain reaction
WME: weighted median estimator

## References

[1] Rothe C, Schunk M, Sothmann P, Bretzel G, Froeschl G, Wallrauch C, et al. Transmission of 2019-nCoV infection from an asymptomatic contact in Germany. N Engl J Med. 2020;382(10):970–1. 10.1056/NEJMc2001468 32003551 PMC7120970

[2] Bai Y, Yao L, Wei T, Tian F, Jin DY, Chen L, et al. Presumed asymptomatic carrier transmission of COVID-19. JAMA. 2020;323(14):1406–7. 10.1001/jama.2020.2565 32083643 PMC7042844

[3] Fan S, He P, Guan J, Song W, Zhi H, Wang L. No association between interleukin-18 levels and risk of cardiovascular disease: a Mendelian randomization study. Hereditas. 2020;157(1):12. 10.1186/s41065-020-00121-5 32264954 PMC7140355

[4] Yang J, Zheng Y, Gou X, Pu K, Chen Z, Guo Q, et al. Prevalence of comorbidities and its effects in patients infected with SARS-CoV-2: a systematic review and meta-analysis. Int J Infect Dis. 2020;94:91–5. 10.1016/j.ijid.2020.03.017 32173574 PMC7194638

[5] Garg S, Kim L, Whitaker M, O’Halloran A, Cummings C, Holstein R, et al. Hospitalization rates and characteristics of patients hospitalized with laboratory-confirmed coronavirus disease 2019—COVID-NET, 14 states, March 1–30, 2020. MMWR Morb Mortal Wkly Rep. 2020;69(15):458–64. 10.15585/mmwr.mm6915e3 32298251 PMC7755063

[6] Richardson S, Hirsch JS, Narasimhan M, Crawford JM, McGinn T, Davidson KW, et al. Presenting characteristics, comorbidities, and outcomes among 5700 patients hospitalized with COVID-19 in the New York City area. JAMA. 2020;323(20):2052–9. 10.1001/jama.2020.6775 32320003 PMC7177629

[7] Akalin E, Azzi Y, Bartash R, Seethamraju H, Parides M, Hemmige V, et al. Covid-19 and kidney transplantation. N Engl J Med. 2020;382(25):2475–7. 10.1056/NEJMc2011117 32329975 PMC7200055

[8] Gold JAW, Wong KK, Szablewski CM, Patel PR, Rossow J, da Silva J, et al. Characteristics and clinical outcomes of adult patients hospitalized with COVID-19—Georgia, March 2020. MMWR Morb Mortal Wkly Rep. 2020;69(18):545–50. 10.15585/mmwr.mm6918e1 32379729 PMC7737948

[9] Hirsch JS, Ng JH, Ross DW, Sharma P, Shah HH, Barnett RL, et al. Acute kidney injury in patients hospitalized with COVID-19. Kidney Int. 2020;98(1):209–18. 10.1016/j.kint.2020.05.006 32416116 PMC7229463

[10] Lighter J, Phillips M, Hochman S, Sterling S, Johnson D, Francois F, et al. Obesity in patients younger than 60 years is a risk factor for Covid-19 hospital admission. Clin Infect Dis. 2020;71(15):896–7. 10.1093/cid/ciaa415 32271368 PMC7184372

[11] Hur K, Price CPE, Gray EL, Gulati RK, Maksimoski M, Racette SD, et al. Factors associated with intubation and prolonged intubation in hospitalized patients with COVID-19. Otolaryngol Head Neck Surg. 2020;163(1):170–8. 10.1177/0194599820929640 32423368 PMC7240317

[12] Simonnet A, Chetboun M, Poissy J, Raverdy V, Noulette J, Duhamel A, et al. High prevalence of obesity in severe acute respiratory syndrome coronavirus-2 (SARS-CoV-2) requiring invasive mechanical ventilation. Obesity (Silver Spring). 2020;28(7):1195–9. 10.1002/oby.22831 32271993 PMC7262326

[13] Kalligeros M, Shehadeh F, Mylona EK, Benitez G, Beckwith CG, Chan PA, et al. Association of obesity with disease severity among patients with coronavirus disease 2019. Obesity (Silver Spring). 2020;28(7):1200–4. 10.1002/oby.22859 32352637 PMC7267224

[14] Palaiodimos L, Kokkinidis DG, Li W, Karamanis D, Ognibene J, Arora S, et al. Severe obesity, increasing age and male sex are independently associated with worse in-hospital outcomes, and higher in-hospital mortality, in a cohort of patients with COVID-19 in the Bronx, New York. Metabolism. 2020;108:154262. 10.1016/j.metabol.2020.154262 32422233 PMC7228874

[15] Zhu L, She ZG, Cheng X, Qin JJ, Zhang XJ, Cai J, et al. Association of blood glucose control and outcomes in patients with COVID-19 and pre-existing type 2 diabetes. Cell Metab. 2020;31(6):1068–77.e3. 10.1016/j.cmet.2020.04.021 32369736 PMC7252168

[16] Bode B, Garrett V, Messler J, McFarland R, Crowe J, Booth R, et al. Glycemic characteristics and clinical outcomes of COVID-19 patients hospitalized in the United States. J Diabetes Sci Technol. 2020;14(4):813–21. 10.1177/1932296820924469 32389027 PMC7673150

[17] Chen Y, Yang D, Cheng B, Chen J, Peng A, Yang C, et al. Clinical characteristics and outcomes of patients with diabetes and COVID-19 in association with glucose-lowering medication. Diabetes Care. 2020;43(7):1399–407. 10.2337/dc20-0660 32409498

[18] Fadini GP, Morieri ML, Longato E, Avogaro A. Prevalence and impact of diabetes among people infected with SARS-CoV-2. J Endocrinol Invest. 2020;43(6):867–9. 10.1007/s40618-020-01236-2 32222956 PMC7103097

[19] Pranata R, Huang I, Lim MA, Wahjoepramono EJ, July J. Impact of cerebrovascular and cardiovascular diseases on mortality and severity of COVID-19-systematic review, meta-analysis, and meta-regression. J Stroke Cerebrovasc Dis. 2020;29(8):104949. 10.1016/j.jstrokecerebrovasdis.2020.104949 PMC7221373 32410807

[20] Wang K, Zhang Z, Yu M, Tao Y, Xie M. 15-day mortality and associated risk factors for hospitalized patients with COVID-19 in Wuhan, China: an ambispective observational cohort study. Intensive Care Med. 2020;46(7):1472–4. 10.1007/s00134-020-06047-w 32328724 PMC7176814

[21] Shi S, Qin M, Shen B, Cai Y, Liu T, Yang F, et al. Association of cardiac injury with mortality in hospitalized patients with COVID-19 in Wuhan, China. JAMA Cardiol. 2020;5(7):802–10. 10.1001/jamacardio.2020.0950 32211816 PMC7097841

[22] Martins-Filho PR, Tavares CSS, Santos VS. Factors associated with mortality in patients with COVID-19. A quantitative evidence synthesis of clinical and laboratory data. Eur J Intern Med. 2020;76:97–9. 10.1016/j.ejim.2020.04.043 32345526 PMC7177074

[23] Wang L, He W, Yu X, Hu D, Bao M, Liu H, et al. Coronavirus disease 2019 in elderly patients: characteristics and prognostic factors based on 4-week follow-up. J Infect. 2020;80(6):639–45. 10.1016/j.jinf.2020.03.019 32240670 PMC7118526

[24] Killerby ME, Link-Gelles R, Haight SC, Schrodt CA, England L, Gomes DJ, et al. Characteristics associated with hospitalization among patients with COVID-19—metropolitan Atlanta, Georgia, March–April 2020. MMWR Morb Mortal Wkly Rep. 2020;69(25):790–4. 10.15585/mmwr.mm6925e1 32584797 PMC7316317

[25] Chen R, Liang W, Jiang M, Guan W, Zhan C, Wang T, et al. Risk factors of fatal outcome in hospitalized subjects with coronavirus disease 2019 from a nationwide analysis in China. Chest. 2020;158(1):97–105. 10.1016/j.chest.2020.04.010 32304772 PMC7158802

[26] Williamson EJ, Walker AJ, Bhaskaran K, Bacon S, Bates C, Morton CE, et al. Factors associated with COVID-19-related death using OpenSAFELY. Nature. 2020;584(7821):430–6. 10.1038/s41586-020-2521-4 32640463 PMC7611074

[27] Guan WJ, Liang WH, Zhao Y, Liang HR, Chen ZS, Li YM, et al. Comorbidity and its impact on 1590 patients with COVID-19 in China: a nationwide analysis. Eur Respir J. 2020;55(5):2000547. 10.1183/13993003.00547-2020 32217650 PMC7098485

[28] Myers LC, Parodi SM, Escobar GJ, Liu VX. Characteristics of hospitalized adults with COVID-19 in an integrated health care system in California. JAMA. 2020;323(21):2195–8. 10.1001/jama.2020.7202 32329797 PMC7182961

[29] Zheng Z, Peng F, Xu B, Zhao J, Liu H, Peng J, et al. Risk factors of critical & mortal COVID-19 cases: a systematic literature review and meta-analysis. J Infect. 2020;81(2):e16–25. 10.1016/j.jinf.2020.04.021 32335169 PMC7177098

[30] Centers for Disease and Control Prevention. Evidence used to update the list of underlying medical conditions that increase a person’s risk of severe illness from COVID-19. Atlanta: Centers for Disease Control and Prevention; 2020 [cited 2020 Jul 20]. Available from: https://www.cdc.gov/coronavirus/2019-ncov/need-extra-precautions/evidence-table.html.

[31] Jordan RE, Adab P. Who is most likely to be infected with SARS-CoV-2? Lancet Infect Dis. 2020;20(9):995–6. 10.1016/S1473-3099(20)30395-9 32422197 PMC7228712

[32] Burgess S, Thompson SG. Multivariable Mendelian randomization: the use of pleiotropic genetic variants to estimate causal effects. Am J Epidemiol. 2015;181(4):251–60. 10.1093/aje/kwu283 25632051 PMC4325677

[33] COVID-19 Host Genetics Initiative. The COVID-19 Host Genetics Initiative, a global initiative to elucidate the role of host genetic factors in susceptibility and severity of the SARS-CoV-2 virus pandemic. Eur J Hum Genet. 2020;28(6):715–8. 10.1038/s41431-020-0636-6 32404885 PMC7220587

[34] Onengut-Gumuscu S, Chen WM, Burren O, Cooper NJ, Quinlan AR, Mychaleckyj JC, et al. Fine mapping of type 1 diabetes susceptibility loci and evidence for colocalization of causal variants with lymphoid gene enhancers. Nat Genet. 2015;47(4):381–6. 10.1038/ng.3245 25751624 PMC4380767

[35] Mahajan A, Taliun D, Thurner M, Robertson NR, Torres JM, Rayner NW, et al. Fine-mapping type 2 diabetes loci to single-variant resolution using high-density imputation and islet-specific epigenome maps. Nat Genet. 2018;50(11):1505–13. 10.1038/s41588-018-0241-6 30297969 PMC6287706

[36] Chen J, Spracklen CN, Marenne G, Varshney A, Corbin LJ, Luan J, et al. The trans-ancestral genomic architecture of glycaemic traits. bioRxiv. 2020 7 25. 10.1101/2020.07.23.217646 PMC7610958 34059833

[37] Locke AE, Kahali B, Berndt SI, Justice AE, Pers TH, Day FR, et al. Genetic studies of body mass index yield new insights for obesity biology. Nature. 2015;518(7538):197–206. 10.1038/nature14177 25673413 PMC4382211

[38] Shungin D, Winkler TW, Croteau-Chonka DC, Ferreira T, Locke AE, Magi R, et al. New genetic loci link adipose and insulin biology to body fat distribution. Nature. 2015;518(7538):187–96. 10.1038/nature14132 25673412 PMC4338562

[39] Willer CJ, Schmidt EM, Sengupta S, Peloso GM, Gustafsson S, Kanoni S, et al. Discovery and refinement of loci associated with lipid levels. Nat Genet. 2013;45(11):1274–83. 10.1038/ng.2797 24097068 PMC3838666

[40] Evangelou E, Warren HR, Mosen-Ansorena D, Mifsud B, Pazoki R, Gao H, et al. Genetic analysis of over 1 million people identifies 535 new loci associated with blood pressure traits. Nat Genet. 2018;50(10):1412–25. 10.1038/s41588-018-0205-x 30224653 PMC6284793

[41] Wuttke M, Li Y, Li M, Sieber KB, Feitosa MF, Gorski M, et al. A catalog of genetic loci associated with kidney function from analyses of a million individuals. Nat Genet. 2019;51(6):957–72. 10.1038/s41588-019-0407-x 31152163 PMC6698888

[42] Nelson CP, Goel A, Butterworth AS, Kanoni S, Webb TR, Marouli E, et al. Association analyses based on false discovery rate implicate new loci for coronary artery disease. Nat Genet. 2017;49(9):1385–91. 10.1038/ng.3913 28714975

[43] Malik R, Chauhan G, Traylor M, Sargurupremraj M, Okada Y, Mishra A, et al. Multiancestry genome-wide association study of 520,000 subjects identifies 32 loci associated with stroke and stroke subtypes. Nat Genet. 2018;50(4):524–37. 10.1038/s41588-018-0058-3 29531354 PMC5968830

[44] Han X, Ong JS, An J, Hewitt AW, Gharahkhani P, MacGregor S. Using Mendelian randomization to evaluate the causal relationship between serum C-reactive protein levels and age-related macular degeneration. Eur J Epidemiol. 2020;35(2):139–46. 10.1007/s10654-019-00598-z 31900758

[45] Hemani G, Zheng J, Elsworth B, Wade KH, Haberland V, Baird D, et al. The MR-Base platform supports systematic causal inference across the human phenome. Elife. 2018;7:e34408. 10.7554/eLife.34408 29846171 PMC5976434

[46] Walker VM, Davies NM, Hemani G, Zheng J, Haycock PC, Gaunt TR, et al. Using the MR-Base platform to investigate risk factors and drug targets for thousands of phenotypes. Wellcome Open Res. 2019;4:113. 10.12688/wellcomeopenres.15334.2 31448343 PMC6694718

[47] McCarthy S, Das S, Kretzschmar W, Delaneau O, Wood AR, Teumer A, et al. A reference panel of 64,976 haplotypes for genotype imputation. Nat Genet. 2016;48(10):1279–83. 10.1038/ng.3643 27548312 PMC5388176

[48] 1000 Genomes Project Consortium, Abecasis GR, Auton A, Brooks LD, DePristo MA, Durbin RM, et al. An integrated map of genetic variation from 1,092 human genomes. Nature. 2012;491(7422):56–65. 10.1038/nature11632 23128226 PMC3498066

[49] Kowalski MH, Qian H, Hou Z, Rosen JD, Tapia AL, Shan Y, et al. Use of >100,000 NHLBI Trans-Omics for Precision Medicine (TOPMed) Consortium whole genome sequences improves imputation quality and detection of rare variant associations in admixed African and Hispanic/Latino populations. PLoS Genet. 2019;15(12):e1008500. 10.1371/journal.pgen.1008500 31869403 PMC6953885

[50] Menni C, Valdes AM, Freidin MB, Sudre CH, Nguyen LH, Drew DA, et al. Real-time tracking of self-reported symptoms to predict potential COVID-19. Nat Med. 2020;26(7):1037–40. 10.1038/s41591-020-0916-2 32393804 PMC7751267

[51] Burgess S, Scott RA, Timpson NJ, Davey Smith G, Thompson SG, EPIC-InterAct Consortium. Using published data in Mendelian randomization: a blueprint for efficient identification of causal risk factors. Eur J Epidemiol. 2015;30(7):543–52. 10.1007/s10654-015-0011-z 25773750 PMC4516908

[52] Bowden J, Del Greco MF, Minelli C, Davey Smith G, Sheehan N, Thompson J. A framework for the investigation of pleiotropy in two-sample summary data Mendelian randomization. Stat Med. 2017;36(11):1783–802. 10.1002/sim.7221 28114746 PMC5434863

[53] Bowden J, Davey Smith G, Haycock PC, Burgess S. Consistent estimation in Mendelian randomization with some invalid instruments using a weighted median estimator. Genet Epidemiol. 2016;40(4):304–14. 10.1002/gepi.21965 27061298 PMC4849733

[54] Bowden J, Davey Smith G, Burgess S. Mendelian randomization with invalid instruments: effect estimation and bias detection through Egger regression. Int J Epidemiol. 2015;44(2):512–25. 10.1093/ije/dyv080 26050253 PMC4469799

[55] Hartwig FP, Davey Smith G, Bowden J. Robust inference in summary data Mendelian randomization via the zero modal pleiotropy assumption. Int J Epidemiol. 2017;46(6):1985–98. 10.1093/ije/dyx102 29040600 PMC5837715

[56] Hwang LD, Lawlor DA, Freathy RM, Evans DM, Warrington NM. Using a two-sample Mendelian randomization design to investigate a possible causal effect of maternal lipid concentrations on offspring birth weight. Int J Epidemiol. 2019;48(5):1457–67. 10.1093/ije/dyz160 31335958 PMC6857765

[57] Verbanck M, Chen CY, Neale B, Do R. Detection of widespread horizontal pleiotropy in causal relationships inferred from Mendelian randomization between complex traits and diseases. Nat Genet. 2018;50(5):693–8. 10.1038/s41588-018-0099-7 29686387 PMC6083837

[58] Sanderson E, Davey Smith G, Windmeijer F, Bowden J. An examination of multivariable Mendelian randomization in the single-sample and two-sample summary data settings. Int J Epidemiol. 2019;48(3):713–27. 10.1093/ije/dyy262 30535378 PMC6734942

[59] Bycroft C, Freeman C, Petkova D, Band G, Elliott LT, Sharp K, et al. The UK Biobank resource with deep phenotyping and genomic data. Nature. 2018;562(7726):203–9. 10.1038/s41586-018-0579-z 30305743 PMC6786975

[60] Spyropoulos AC, Weitz JI. Hospitalized COVID-19 patients and venous thromboembolism: a perfect storm. Circulation. 2020;142(2):129–32. 10.1161/CIRCULATIONAHA.120.048020 32658609

[61] Klarin D, Busenkell E, Judy R, Lynch J, Levin M, Haessler J, et al. Genome-wide association analysis of venous thromboembolism identifies new risk loci and genetic overlap with arterial vascular disease. Nat Genet. 2019;51(11):1574–9. 10.1038/s41588-019-0519-3 31676865 PMC6858581

[62] Ponsford MJ, Gkatzionis A, Walker VM, Grant AJ, Wootton RE, Moore LSP, et al. Cardiometabolic traits, sepsis, and severe COVID-19: A Mendelian randomization investigation. Circulation. 2020;142(18):1791–3. 10.1161/CIRCULATIONAHA.120.050753 32966752 PMC7594537

[63] Freuer D, Linseisen J, Meisinger C. Impact of body composition on COVID-19 susceptibility and severity: a two-sample multivariable Mendelian randomization study. medRxiv. 2020 10 24. 10.1101/2020.07.14.20153825 PMC7900753 33631142

[64] Zhang X, Li X, Sun Z, He Y, Xu W, Campbell H, et al. Physical activity and COVID-19: an observational and Mendelian randomisation study. J Glob Health. 2020;10(2):020514. 10.7189/jogh-10-020514 33312507 PMC7719276

[65] Griffith GJ, Morris TT, Tudball MJ, Herbert A, Mancano G, Pike L, et al. Collider bias undermines our understanding of COVID-19 disease risk and severity. Nat Commun. 2020;11(1):5749. 10.1038/s41467-020-19478-2 33184277 PMC7665028

[66] Thomas LE, Bonow RO, Pencina MJ. Understanding observational treatment comparisons in the setting of coronavirus disease 2019 (COVID-19). JAMA Cardiol. 2020;5(9):988–90. 10.1001/jamacardio.2020.1874 32936260

[67] Burgess S, Davies NM, Thompson SG. Bias due to participant overlap in two-sample Mendelian randomization. Genet Epidemiol. 2016;40(7):597–608. 10.1002/gepi.21998 27625185 PMC5082560

[68] Chen T, Wu D, Chen H, Yan W, Yang D, Chen G, et al. Clinical characteristics of 113 deceased patients with coronavirus disease 2019: retrospective study. BMJ. 2020;368:m1091. 10.1136/bmj.m1091 32217556 PMC7190011

[69] Thompson BT, Chambers RC, Liu KD. Acute respiratory distress syndrome. N Engl J Med. 2017;377(6):562–72. 10.1056/NEJMra1608077 28792873

[70] Yancy CW. COVID-19 and African Americans. JAMA. 2020;323(19):1891–2. 10.1001/jama.2020.6548 32293639

[71] Stokes EK, Zambrano LD, Anderson KN, Marder EP, Raz KM, El Burai Felix S, et al. Coronavirus disease 2019 case surveillance—United States, January 22–May 30, 2020. MMWR Morb Mortal Wkly Rep. 2020;69(24):759–65. 10.15585/mmwr.mm6924e2 32555134 PMC7302472

[72] Millett GA, Jones AT, Benkeser D, Baral S, Mercer L, Beyrer C, et al. Assessing differential impacts of COVID-19 on black communities. Ann Epidemiol. 2020;47:37–44. 10.1016/j.annepidem.2020.05.003 32419766 PMC7224670

[73] Christakis NA, Fowler JH. The spread of obesity in a large social network over 32 years. N Engl J Med. 2007;357(4):370–9. 10.1056/NEJMsa066082 17652652

[74] Lakerveld J, Mackenbach J. The upstream determinants of adult obesity. Obes Facts. 2017;10(3):216–22. 10.1159/000471489 28564658 PMC5644962

