# Supplementary material for: Cardiometabolic risk factors for COVID-19 susceptibility and severity: A Mendelian randomization analysis
Source: PLoS Med. 2021 Mar 4;18(3):e1003553. doi: 10.1371/journal.pmed.1003553 (PMC7971850; doi:10.1371/journal.pmed.1003553)
Supplement: S1 Figs — (PDF) [file pmed.1003553.s002.pdf]

Fig A. Forest plot of MR effect estimates and 95% confidence interval for each exposure and the two main outcomes analyzed, excluding all variants with F-statistics below 10 with a LD clumping threshold of $r^2<0.001$. See Figure 1 for exposure units.

COVID-19 Susceptibility: COVID positive vs. population controls

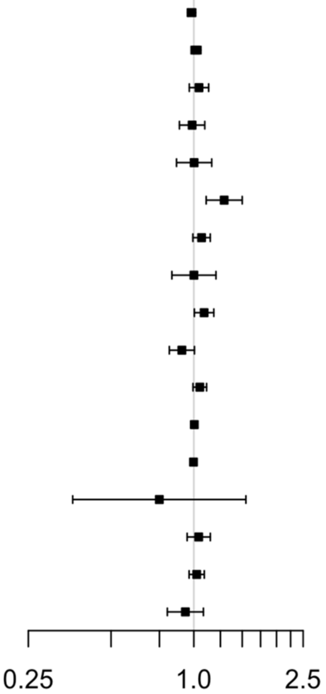

Exposure	OR (95% CI)	P-value
Type 1 Diabetes	0.98 (0.95 to 1.02)	0.29
Type 2 Diabetes	1.02 (0.98 to 1.06)	0.37
Hemoglobin A1c	1.04 (0.96 to 1.13)	0.29
Fasting Glucose – BMI Adjusted	0.99 (0.89 to 1.10)	0.80
Fasting Insulin – BMI Adjusted	1.00 (0.87 to 1.16)	0.97
Body Mass Index	1.29 (1.11 to 1.50)	9.3×10^{-4}
C-reactive Protein	1.07 (0.99 to 1.15)	0.08
Waist-hip Ratio – BMI Adjusted	1.00 (0.83 to 1.20)	0.99
Low Density Lipoprotein	1.09 (1.01 to 1.18)	0.04
High Density Lipoprotein	0.91 (0.81 to 1.01)	0.06
Triglycerides	1.05 (0.99 to 1.11)	0.08
Systolic Blood Pressure	1.00 (0.99 to 1.02)	0.46
Diastolic Blood Pressure	1.00 (0.98 to 1.02)	0.80
Creatinine-based eGFR	0.75 (0.36 to 1.55)	0.44
Chronic Kidney Disease	1.04 (0.95 to 1.15)	0.40
Coronary Artery Disease	1.02 (0.96 to 1.09)	0.45
Any Stroke	0.93 (0.80 to 1.08)	0.36

COVID-19 Severity: Hospitalization vs. population controls

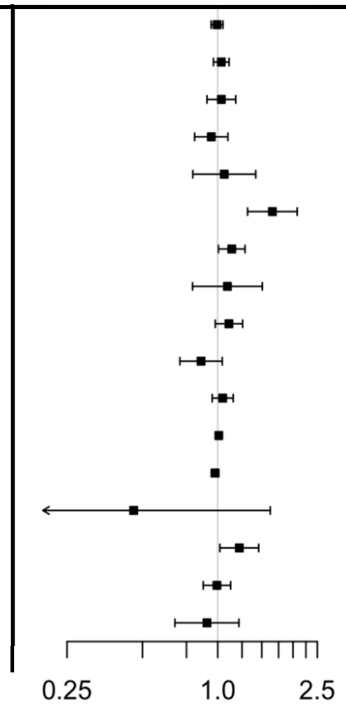

Exposure	OR (95% CI)	P-value
Type 1 Diabetes	0.99 (0.94 to 1.05)	0.82
Type 2 Diabetes	1.03 (0.96 to 1.11)	0.37
Hemoglobin A1c	1.03 (0.91 to 1.18)	0.62
Fasting Glucose – BMI Adjusted	0.94 (0.81 to 1.10)	0.45
Fasting Insulin – BMI Adjusted	1.06 (0.80 to 1.42)	0.68
Body Mass Index	1.65 (1.32 to 2.08)	1.6×10^{-5}
C-reactive Protein	1.14 (1.01 to 1.29)	0.04
Waist-hip Ratio – BMI Adjusted	1.09 (0.79 to 1.51)	0.59
Low Density Lipoprotein	1.11 (0.98 to 1.26)	0.11
High Density Lipoprotein	0.86 (0.71 to 1.04)	0.12
Triglycerides	1.05 (0.95 to 1.15)	0.36
Systolic Blood Pressure	1.01 (0.99 to 1.03)	0.36
Diastolic Blood Pressure	0.98 (0.94 to 1.01)	0.15
Creatinine-based eGFR	0.46 (0.13 to 1.62)	0.23
Chronic Kidney Disease	1.22 (1.02 to 1.46)	0.03
Coronary Artery Disease	0.99 (0.87 to 1.13)	0.91
Any Stroke	0.91 (0.67 to 1.22)	0.51

Fig B. The relationship between the SNP effects on the exposure, Body Mass Index, and the outcome, COVID-19 vs. population controls across multiple MR methods.

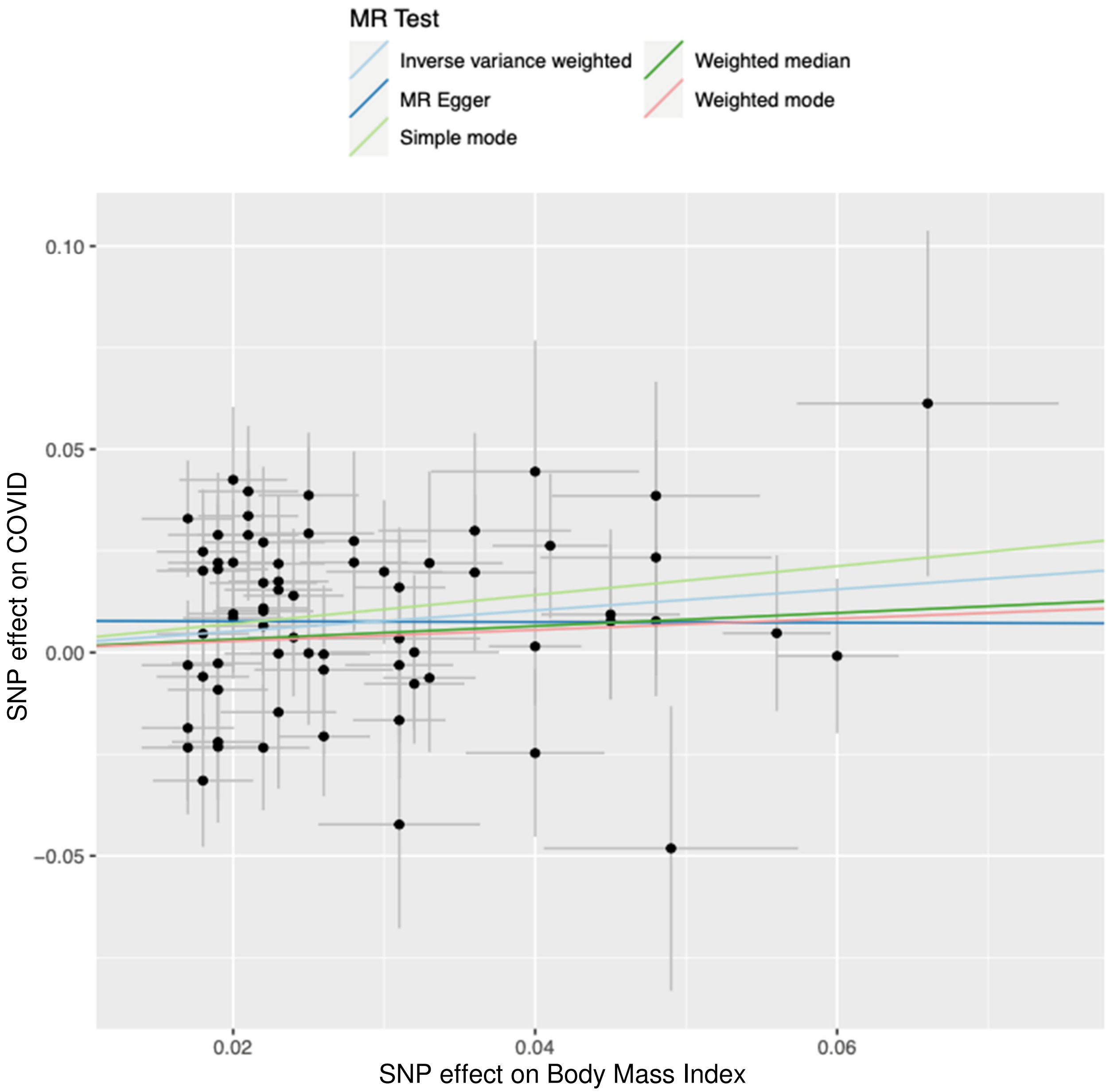

Fig C. Funnel plot of the Body Mass Index instrument precision versus the MR effect estimates on the outcome, COVID-19 vs. population controls.

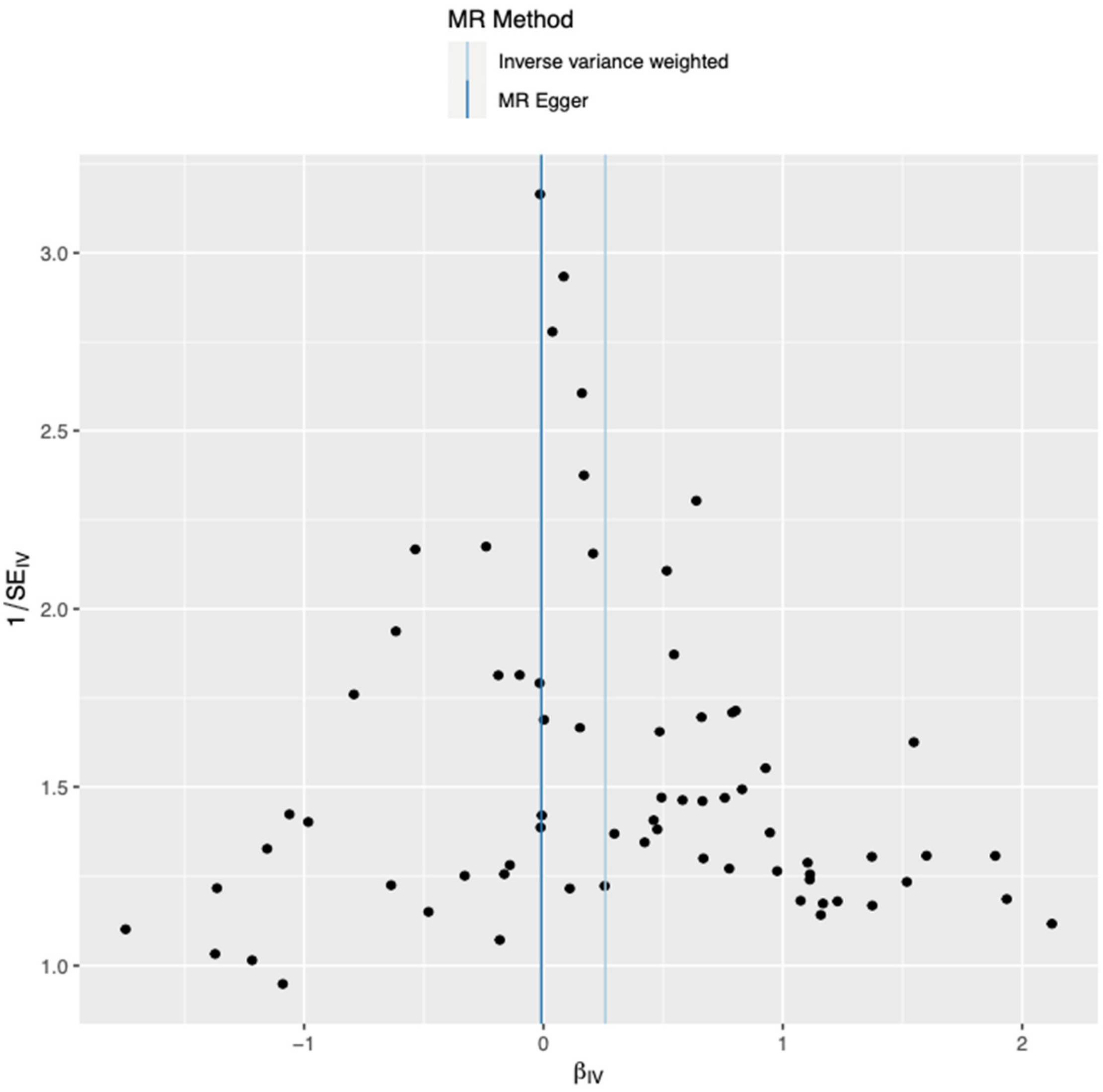

SNP effect on Body Mass Index

Fig D. Leave-one-out MR sensitivity analysis of Body Mass Index on the outcome, COVID-19 vs. population controls, estimating the IVW effect estimate after each SNP is removed.

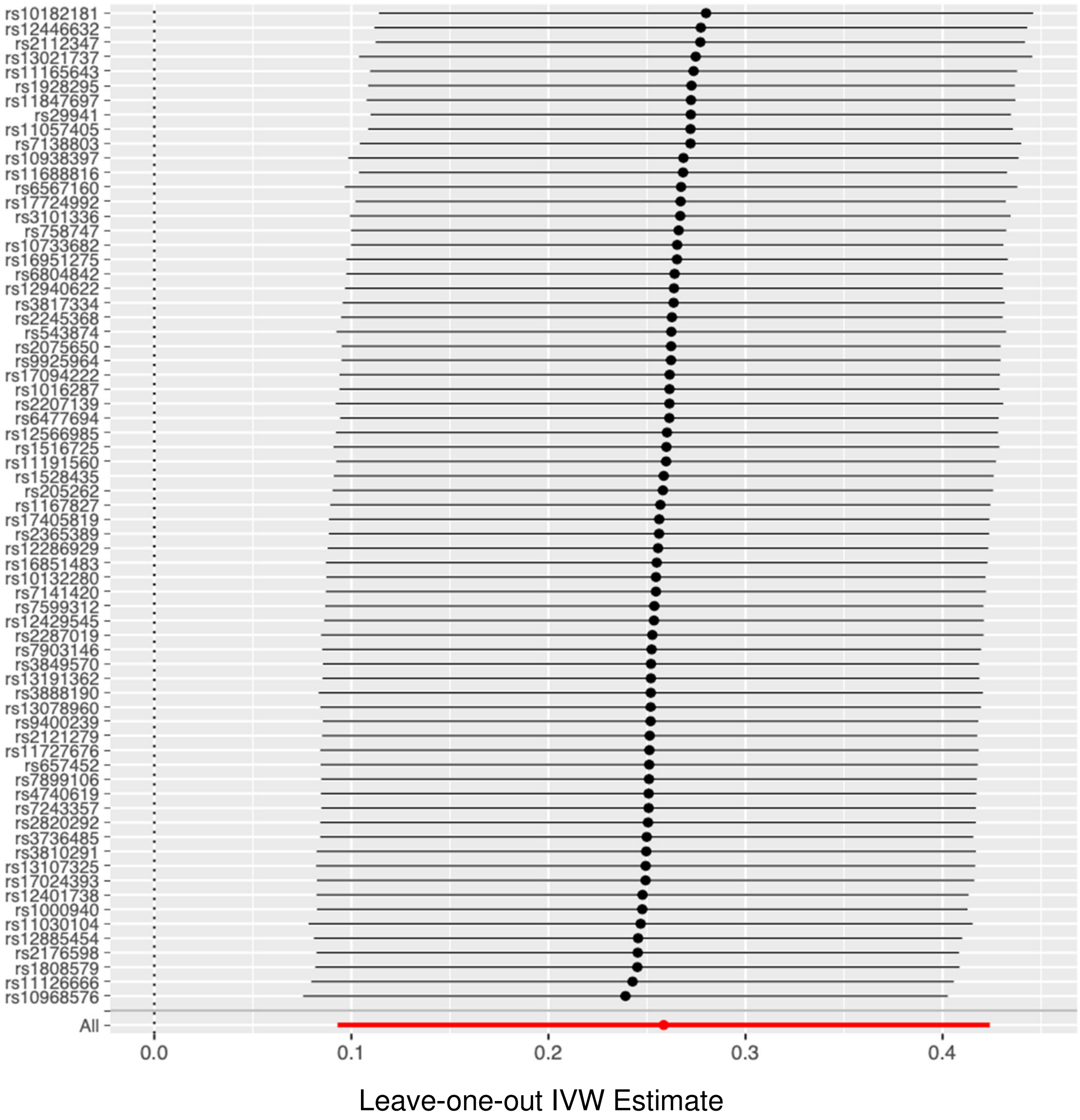

Fig E. The relationship between the SNP effects on the exposure, Body Mass Index, and the outcome, COVID-19 hospitalization vs. population controls across multiple MR methods.

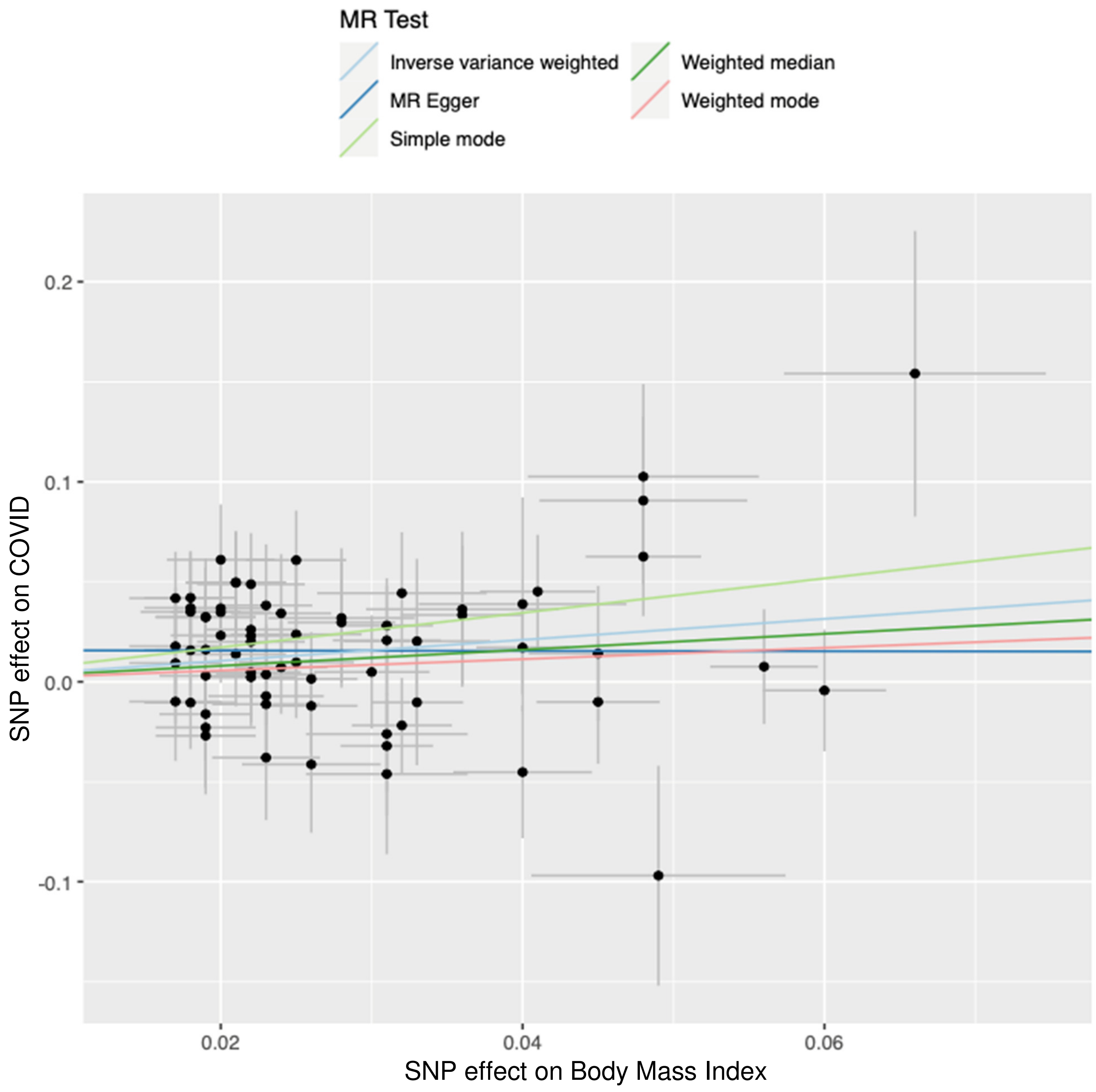

Fig F. Funnel plot of the Body Mass Index instrument precision versus the MR effect estimates on the outcome, COVID-19 hospitalization vs. population controls.

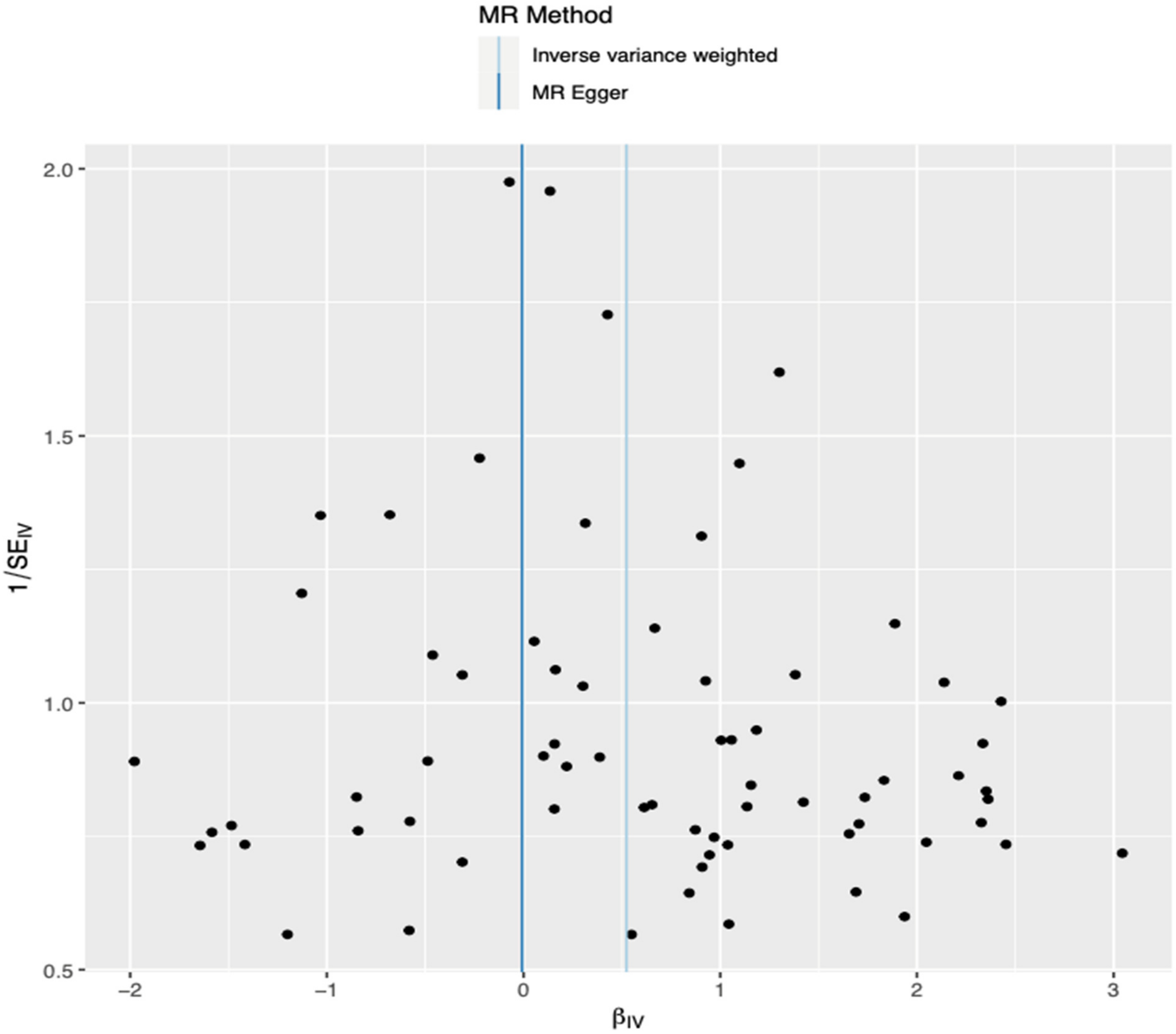

SNP effect on Body Mass Index

Fig G. Leave-one-out MR sensitivity analysis of Body Mass Index on the outcome, COVID-19 hospitalization vs. population controls, estimating the IVW estimate after each SNP is removed.

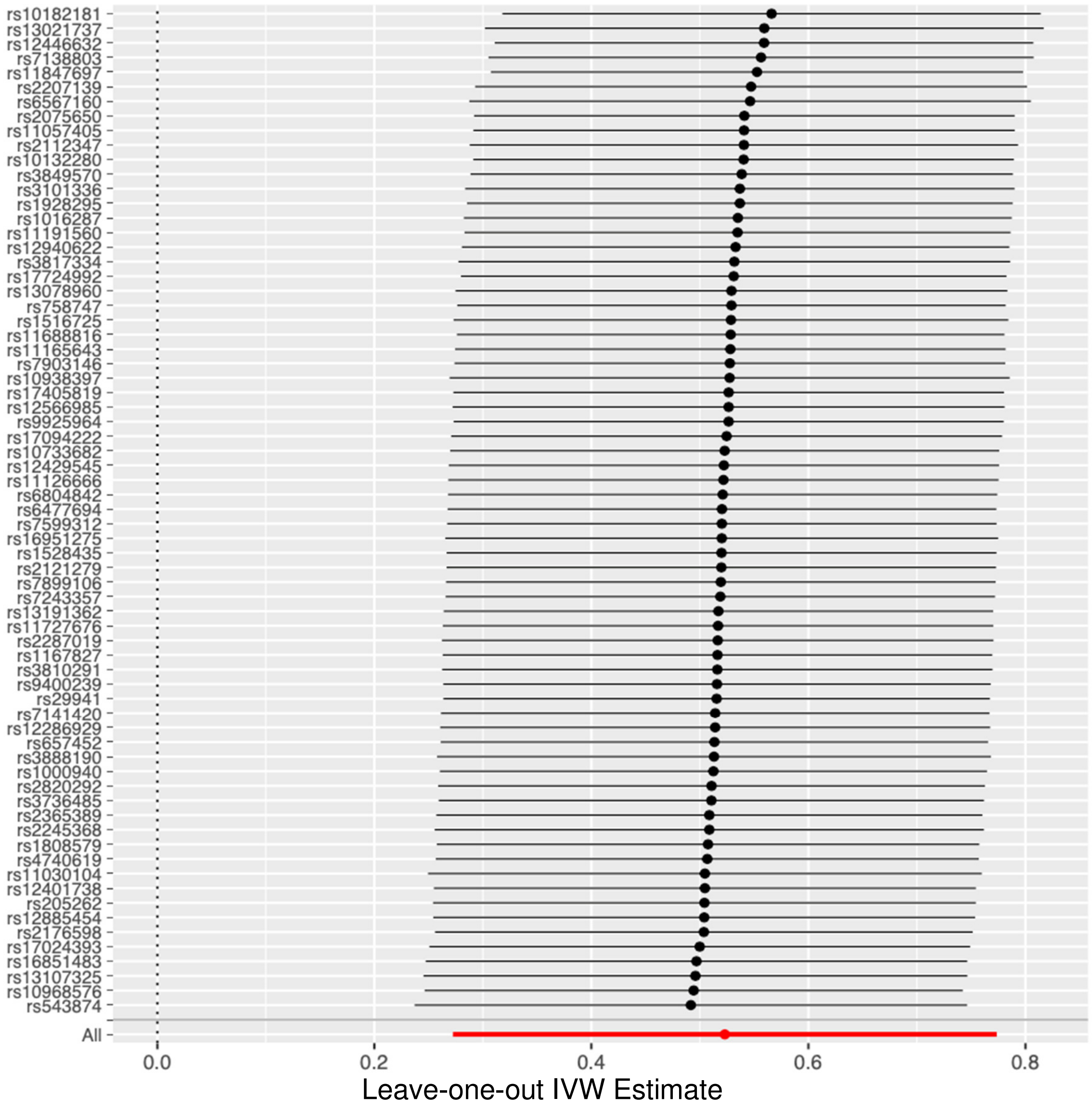

Fig H. Forest plot of MR effect estimates and 95% confidence intervals for each exposure and critical respiratory illness vs. not hospitalized with COVID-19. See Figure 1 for exposure units.

Critical respiratory illness with COVID-19 vs. not hospitalized with COVID-19

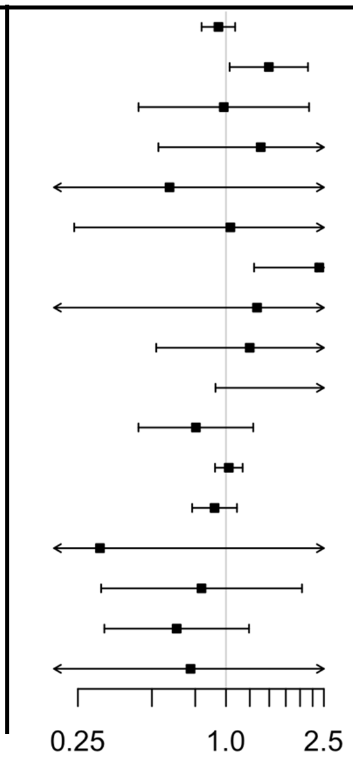

Exposure	OR (95% CI)	P-value
Type 1 Diabetes	0.93 (0.80 to 1.09)	0.38
Type 2 Diabetes	1.49 (1.04 to 2.15)	0.03
Hemoglobin A1c	0.98 (0.44 to 2.17)	0.96
Fasting Glucose – BMI Adjusted	1.38 (0.53 to 3.61)	0.51
Fasting Insulin – BMI Adjusted	0.59 (0.12 to 2.81)	0.51
Body Mass Index	1.04 (0.24 to 4.50)	0.96
C-reactive Protein	2.39 (1.30 to 4.41)	5.1×10^{-3}
Waist-hip Ratio – BMI Adjusted	1.34 (0.18 to 9.86)	0.78
Low Density Lipoprotein	1.25 (0.52 to 3.00)	0.62
High Density Lipoprotein	2.58 (0.91 to 7.32)	0.08
Triglycerides	0.75 (0.44 to 1.29)	0.30
Systolic Blood Pressure	1.03 (0.90 to 1.17)	0.69
Diastolic Blood Pressure	0.90 (0.73 to 1.11)	0.32
Creatinine-based eGFR	0.31 (0.00 to 304.82)	0.74
Chronic Kidney Disease	0.80 (0.31 to 2.04)	0.63
Coronary Artery Disease	0.63 (0.32 to 1.24)	0.18
Any Stroke	0.72 (0.17 to 3.11)	0.66

Fig I. Forest plot of MR effect estimates and 95% confidence intervals for each exposure and critical respiratory illness with COVID-19 vs. population controls. See Figure 1 for exposure units.

Critical respiratory illness with COVID-19 vs. population controls

Exposure	OR (95% CI)	P-value
Type 1 Diabetes	0.97 (0.94 to 1.01)	0.14
Type 2 Diabetes	1.03 (0.95 to 1.11)	0.51
Hemoglobin A1c	1.05 (0.90 to 1.23)	0.50
Fasting Glucose – BMI Adjusted	1.05 (0.87 to 1.27)	0.61
Fasting Insulin – BMI Adjusted	0.84 (0.61 to 1.15)	0.27
Body Mass Index	1.67 (1.24 to 2.26)	7.7×10^{-4}
C-reactive Protein	1.11 (0.97 to 1.26)	0.13
Waist-hip Ratio – BMI Adjusted	0.89 (0.61 to 1.30)	0.55
Low Density Lipoprotein	1.09 (0.93 to 1.27)	0.31
High Density Lipoprotein	0.97 (0.78 to 1.19)	0.74
Triglycerides	0.95 (0.85 to 1.06)	0.36
Systolic Blood Pressure	1.01 (0.98 to 1.04)	0.41
Diastolic Blood Pressure	1.00 (0.96 to 1.05)	0.83
Creatinine-based eGFR	0.45 (0.10 to 2.17)	0.32
Chronic Kidney Disease	1.15 (0.93 to 1.42)	0.20
Coronary Artery Disease	0.91 (0.78 to 1.05)	0.20
Any Stroke	0.92 (0.63 to 1.36)	0.69

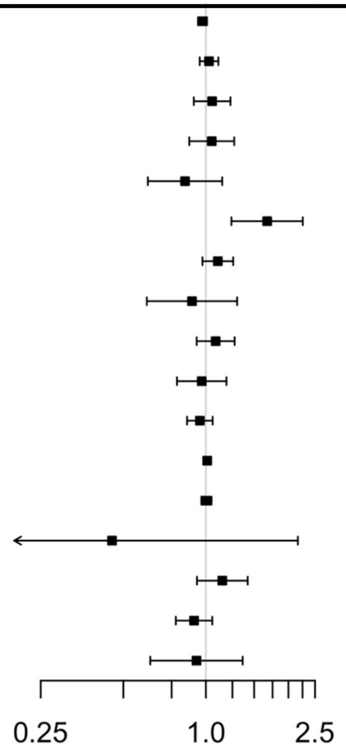

Fig J. Forest plot of MR effect estimates and 95% confidence intervals for each exposure and hospitalized vs. not hospitalized with COVID-19. See Figure 1 for exposure units.

Hospitalized with COVID-19 vs. not hospitalized with COVID-19

Exposure	OR (95% CI)	P-value
Type 1 Diabetes	1.00 (0.96 to 1.05)	0.99
Type 2 Diabetes	1.05 (0.96 to 1.16)	0.29
Hemoglobin A1c	1.14 (0.91 to 1.44)	0.26
Fasting Glucose – BMI Adjusted	0.85 (0.66 to 1.10)	0.23
Fasting Insulin – BMI Adjusted	1.12 (0.75 to 1.65)	0.58
Body Mass Index	1.50 (0.95 to 2.37)	0.08
C-reactive Protein	1.30 (1.10 to 1.54)	2.0×10^{-3}
Waist-hip Ratio – BMI Adjusted	1.19 (0.71 to 2.00)	0.51
Low Density Lipoprotein	1.03 (0.83 to 1.27)	0.80
High Density Lipoprotein	0.88 (0.67 to 1.16)	0.36
Triglycerides	1.15 (0.99 to 1.33)	0.06
Systolic Blood Pressure	1.00 (0.96 to 1.03)	0.85
Diastolic Blood Pressure	1.00 (0.95 to 1.06)	0.95
Creatinine-based eGFR	1.75 (0.27 to 11.48)	0.56
Chronic Kidney Disease	1.03 (0.76 to 1.40)	0.84
Coronary Artery Disease	1.13 (0.91 to 1.39)	0.27
Any Stroke	1.00 (0.59 to 1.72)	0.99

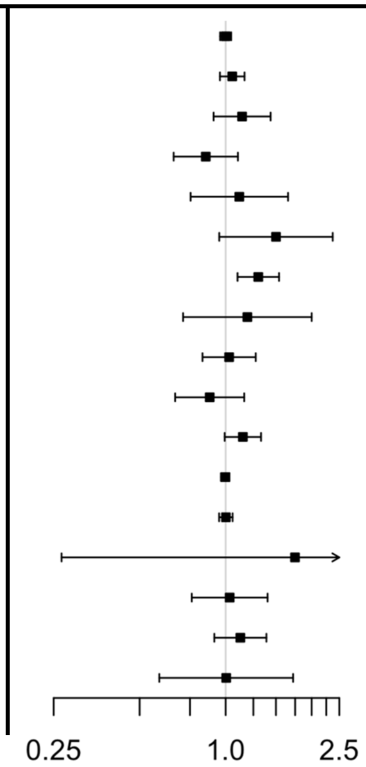

Fig K. Forest plot of MR effect estimates and 95% confidence intervals for each exposure and COVID-19 positive vs. COVID-19 negative. See Figure 1 for exposure units.

COVID-19 positive vs. lab/self-reported COVID-19 negative

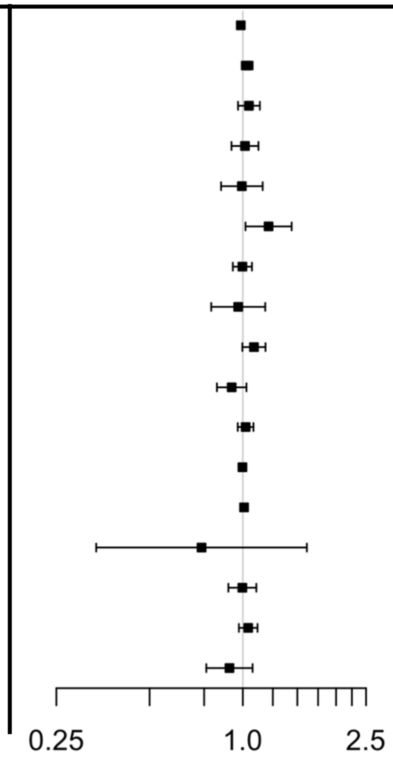

Exposure	OR (95% CI)	P-value
Type 1 Diabetes	0.98 (0.97 to 1.00)	0.13
Type 2 Diabetes	1.03 (0.99 to 1.08)	0.12
Hemoglobin A1c	1.05 (0.96 to 1.13)	0.27
Fasting Glucose – BMI Adjusted	1.02 (0.92 to 1.12)	0.76
Fasting Insulin – BMI Adjusted	0.99 (0.85 to 1.16)	0.93
Body Mass Index	1.21 (1.02 to 1.44)	0.03
C-reactive Protein	1.00 (0.93 to 1.07)	0.93
Waist-hip Ratio – BMI Adjusted	0.97 (0.79 to 1.18)	0.74
Low Density Lipoprotein	1.09 (1.00 to 1.18)	0.06
High Density Lipoprotein	0.92 (0.82 to 1.03)	0.14
Triglycerides	1.02 (0.96 to 1.08)	0.49
Systolic Blood Pressure	1.00 (0.98 to 1.01)	0.66
Diastolic Blood Pressure	1.01 (0.99 to 1.03)	0.41
Creatinine-based eGFR	0.74 (0.34 to 1.61)	0.44
Chronic Kidney Disease	1.00 (0.90 to 1.11)	0.95
Coronary Artery Disease	1.04 (0.97 to 1.12)	0.26
Any Stroke	0.91 (0.76 to 1.08)	0.26

Fig L. Forest plot of MR effect estimates and 95% confidence intervals for each exposure and predicted COVID-19 positive vs. COVID-19 negative. See Figure 1 for exposure units.

Predicted COVID-19 vs. predicted or self-reported COVID-19 negative

Exposure	OR (95% CI)	P-value
Type 1 Diabetes	1.00 (0.97 to 1.03)	0.95
Type 2 Diabetes	0.99 (0.92 to 1.06)	0.73
Hemoglobin A1c	0.87 (0.75 to 1.00)	0.04
Fasting Glucose – BMI Adjusted	0.96 (0.80 to 1.15)	0.66
Fasting Insulin – BMI Adjusted	1.00 (0.76 to 1.31)	0.99
Body Mass Index	0.93 (0.71 to 1.21)	0.60
C-reactive Protein	1.06 (0.94 to 1.19)	0.37
Waist-hip Ratio – BMI Adjusted	0.98 (0.69 to 1.40)	0.93
Low Density Lipoprotein	1.09 (0.95 to 1.26)	0.21
High Density Lipoprotein	0.81 (0.65 to 1.01)	0.06
Triglycerides	1.04 (0.94 to 1.15)	0.42
Systolic Blood Pressure	1.00 (0.98 to 1.02)	0.97
Diastolic Blood Pressure	1.03 (1.00 to 1.07)	0.08
Creatinine-based eGFR	0.62 (0.18 to 2.09)	0.44
Chronic Kidney Disease	0.93 (0.77 to 1.14)	0.50
Coronary Artery Disease	1.04 (0.91 to 1.18)	0.57
Any Stroke	1.12 (0.80 to 1.57)	0.49

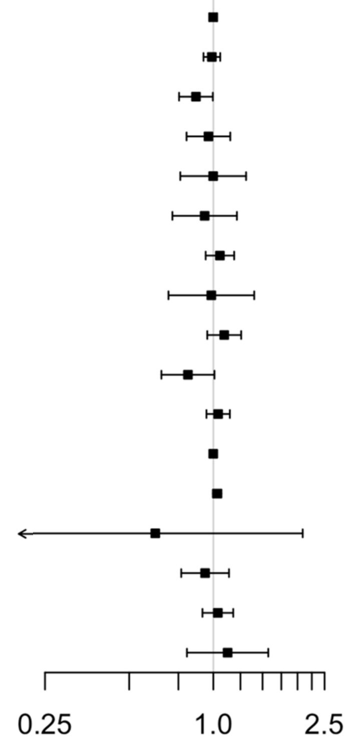

Fig M. The relationship between the SNP effects on the exposure, Body Mass Index, and the outcome, critical respiratory illness vs. no hospitalization with COVID-19 across multiple MR methods.

.

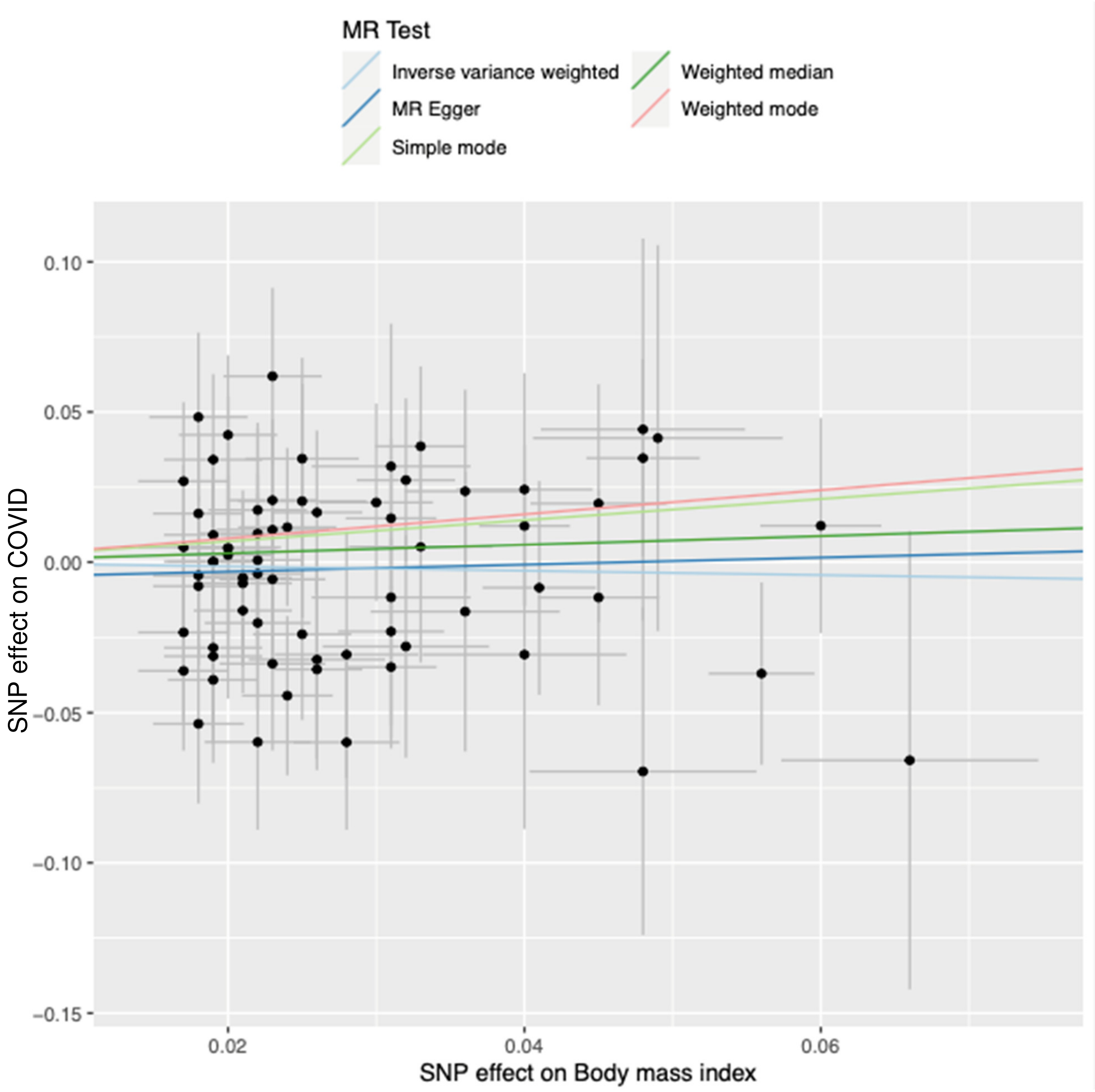

Fig N. The relationship between the SNP effects on the exposure, Body Mass Index, and the outcome, critical respiratory illness with COVID-19 vs. population controls across multiple MR methods.

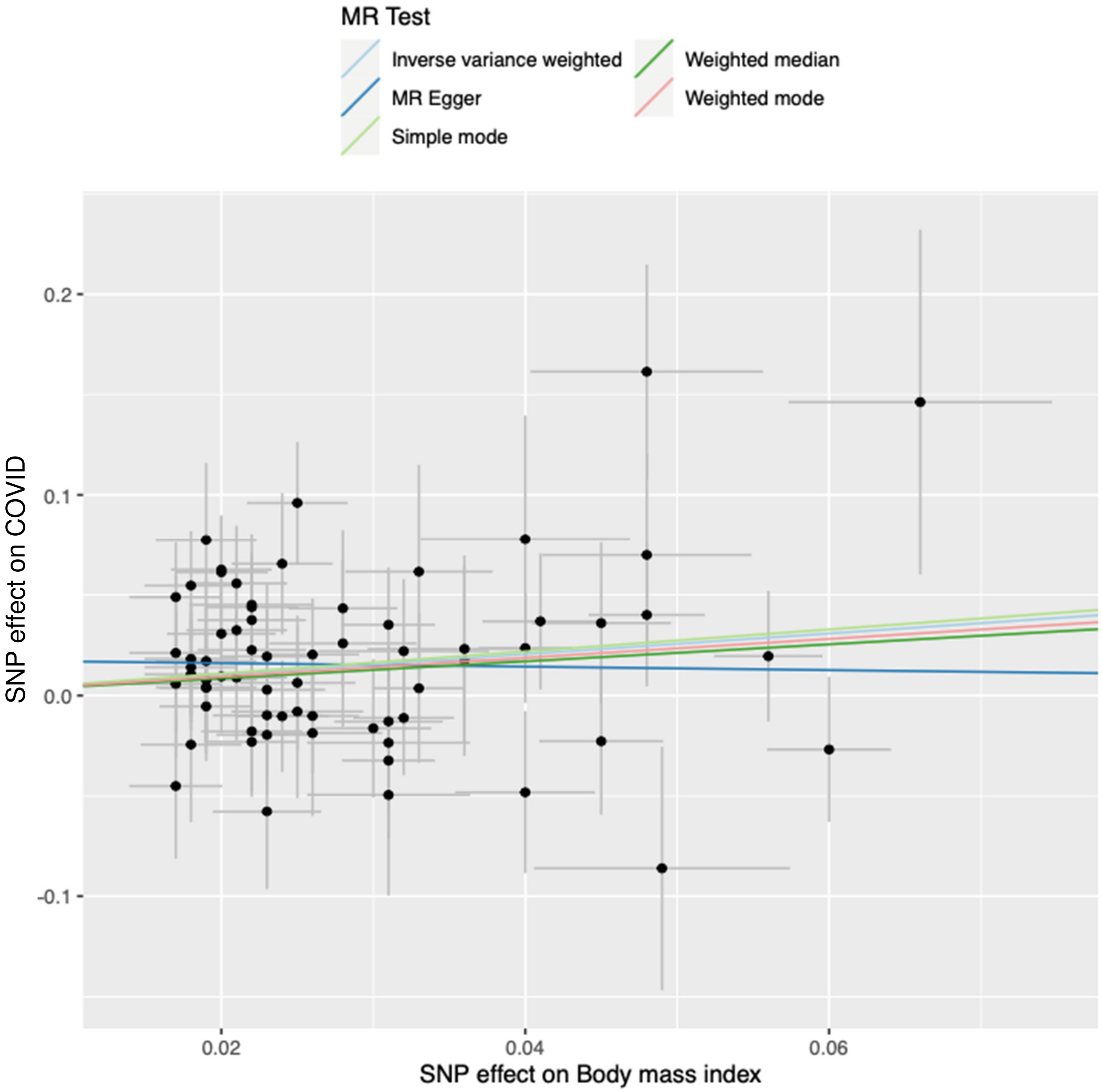

Fig O. The relationship between the SNP effects on the exposure, Body Mass Index, and the outcome, hospitalized with COVID-19 vs. not hospitalized with COVID-19 across multiple MR methods.

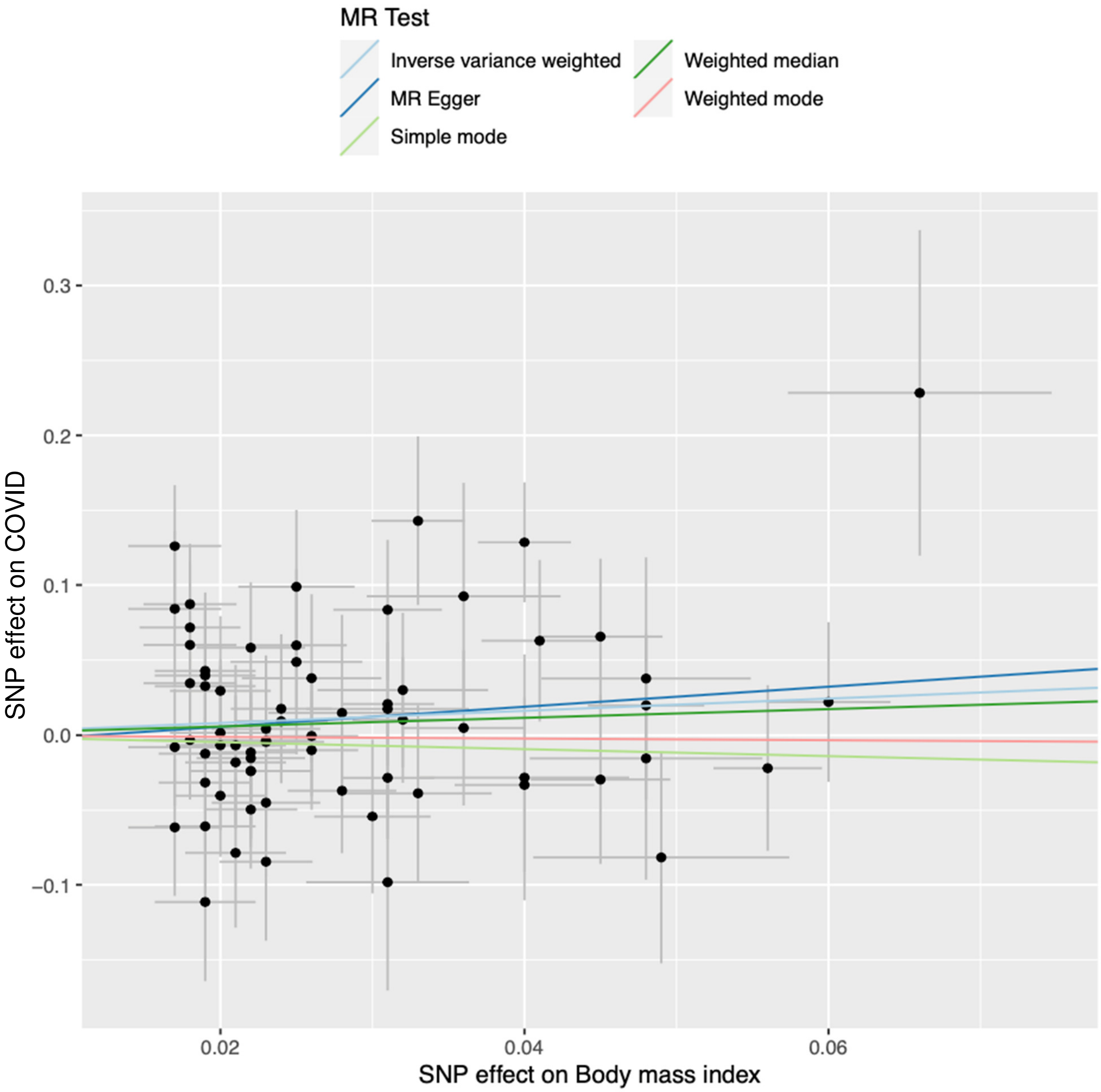

Fig P. The relationship between the SNP effects on the exposure, Body Mass Index, and the outcome, COVID-19 positive vs. lab or self-reported COVID-19 negative across multiple MR methods.

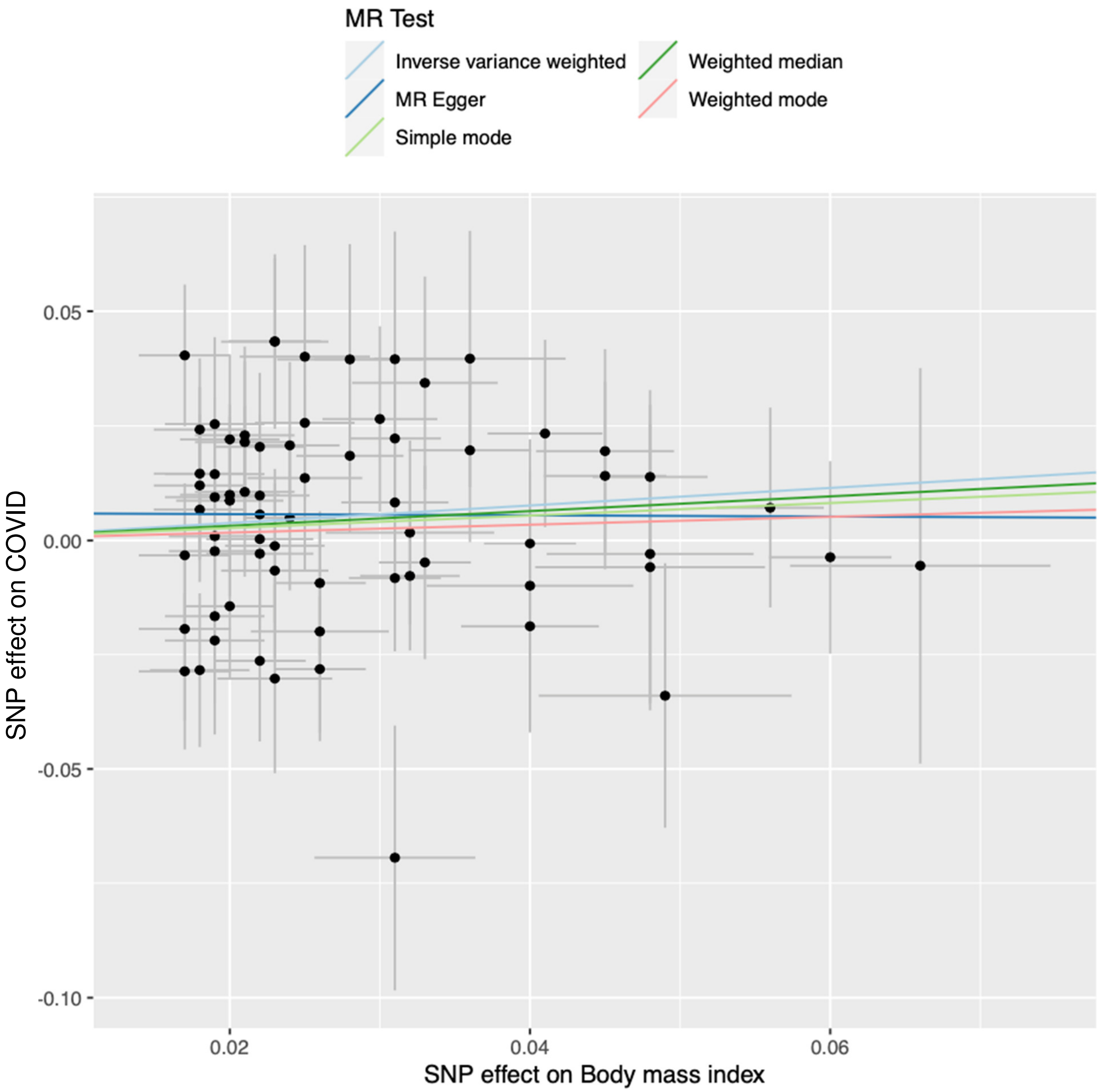

Fig Q. The relationship between the SNP effects on the exposure, Body Mass Index, and the outcome, predicted COVID-19 vs. predicted or self-reported COVID-19 negative across multiple MR methods.

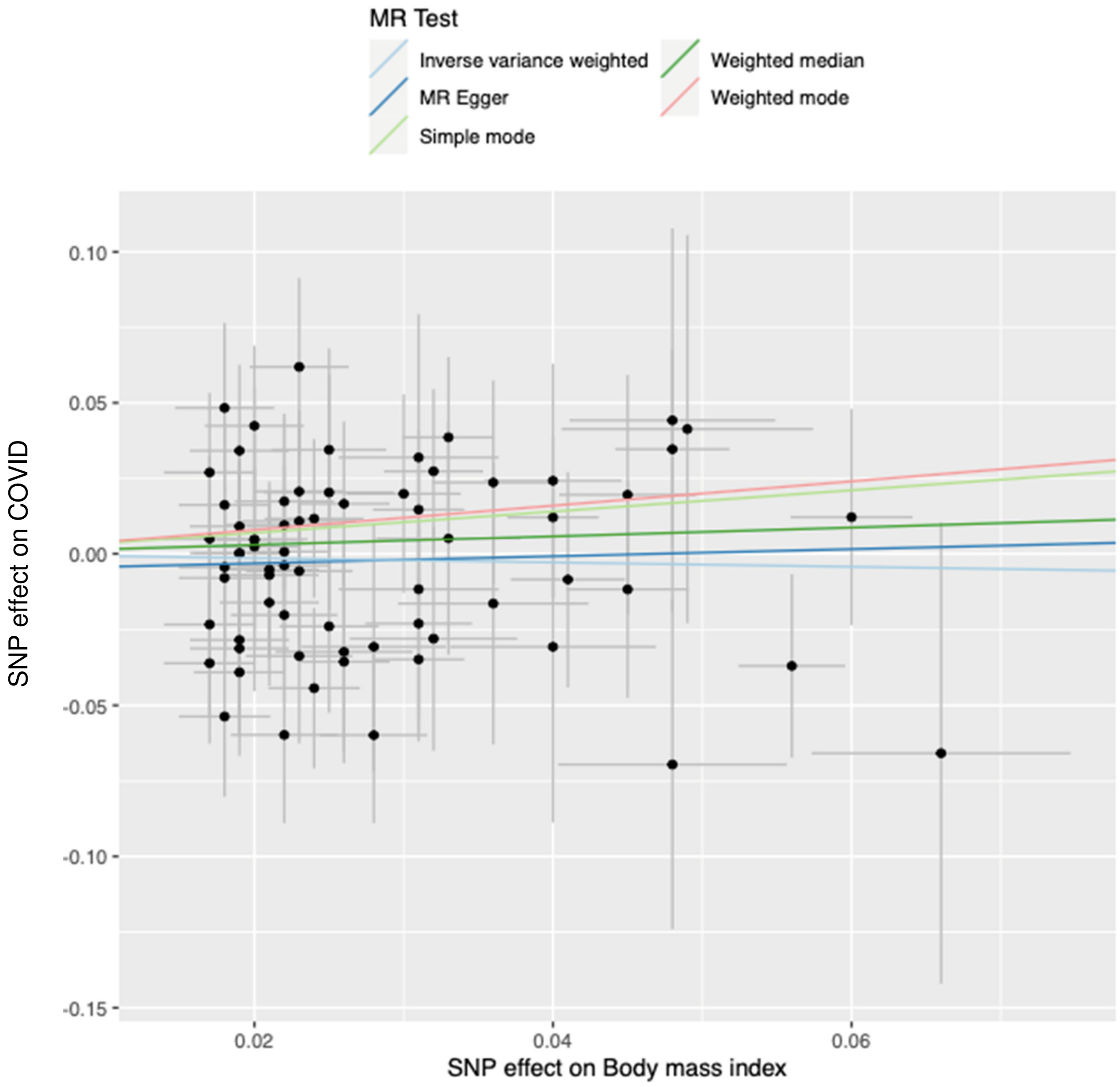

Fig R. Forest plot MR effect estimates and 95% confidence interval for venous thromboembolism across all seven COVID-19 HGI outcomes.

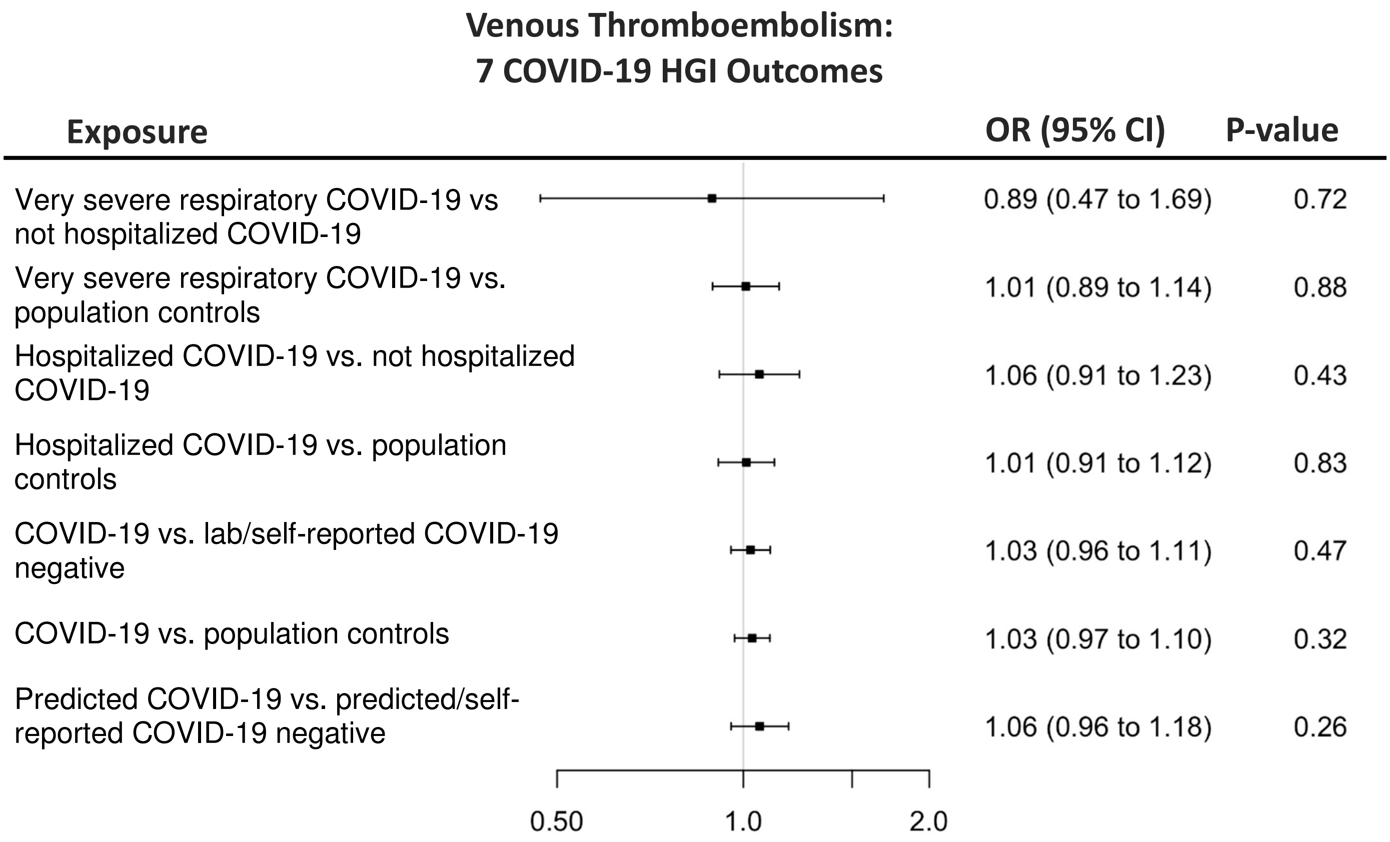

Fig S. Forest plot MR effect estimates and 95% confidence interval for obesity related traits, adjusted and unadjusted for Body Mass Index on COVID-19.

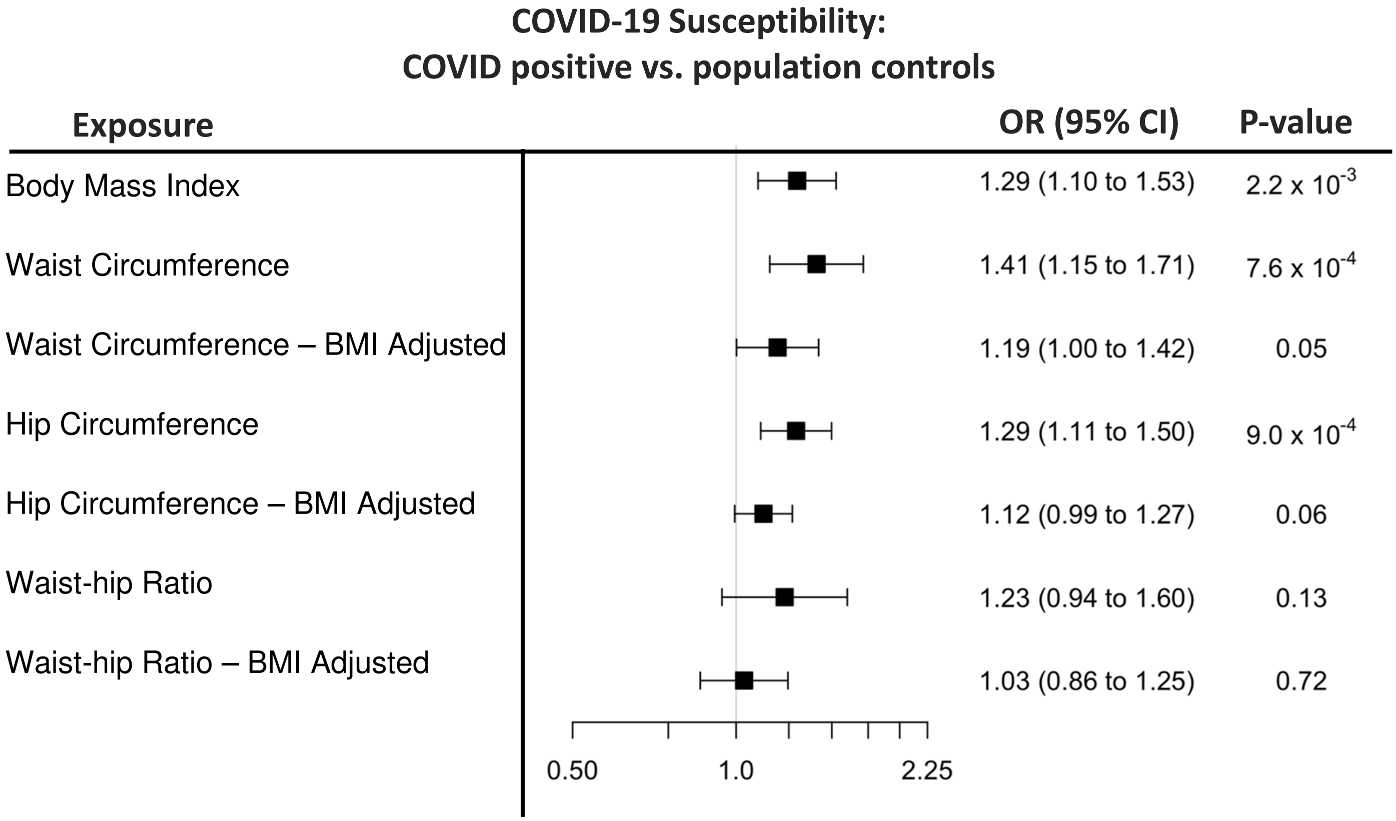

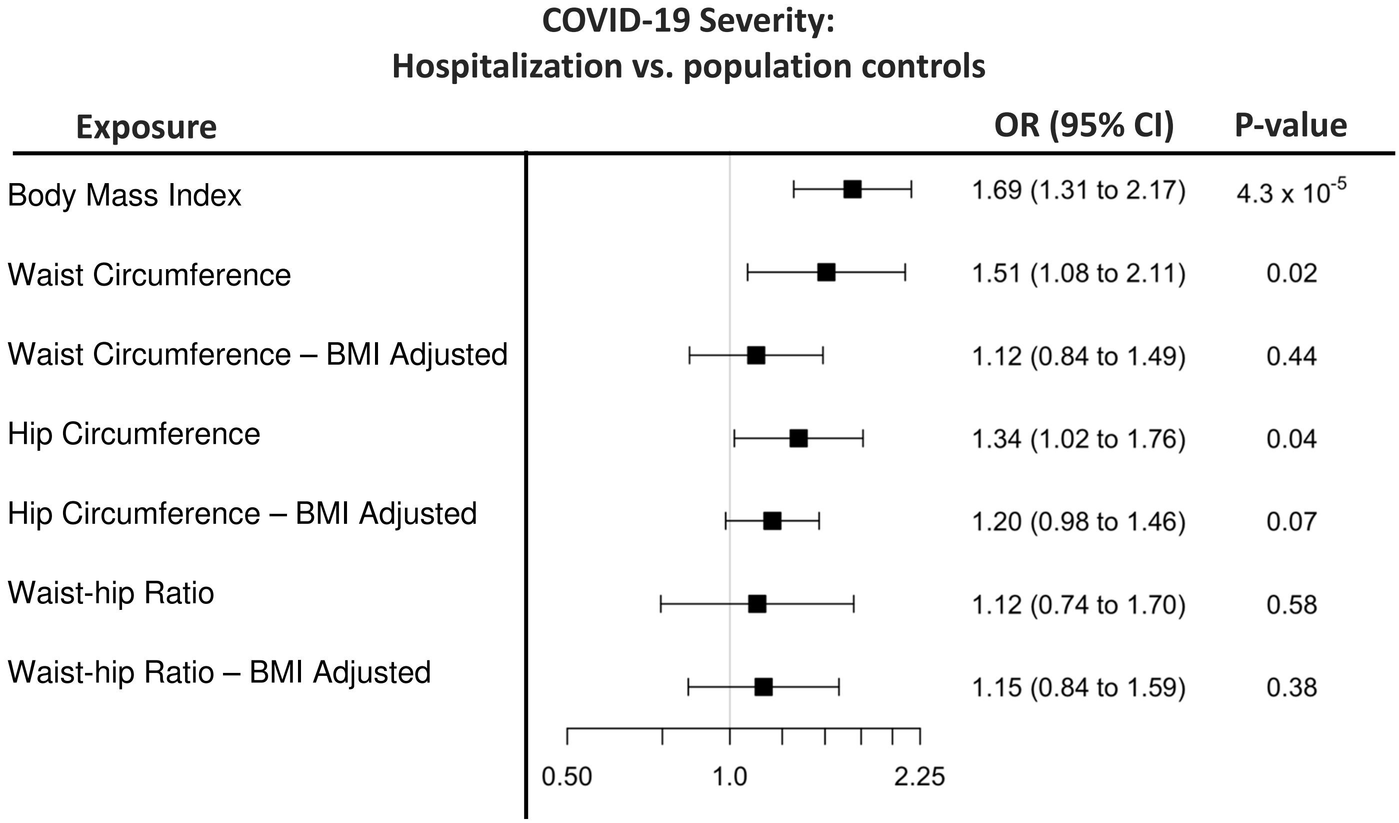

MR: Mendelian randomization; BMI: Body mass index, OR: odds ratio; CI: confidence interval; SNP: single nucleotide polymorphism
