# Supplementary material for: Cardiometabolic risk factors for COVID-19 susceptibility and severity: A Mendelian randomization analysis
Source: PLoS Med. 2021 Mar 4;18(3):e1003553. doi: 10.1371/journal.pmed.1003553 (PMC7971850; doi:10.1371/journal.pmed.1003553)
Supplement: S1 Tables — (DOCX) [file pmed.1003553.s003.docx]

**Table A.** Summary of F-statistics for all instruments used for each trait.

| **Exposure** | **Median** | **Number of SNPs with F-statistic below 10** | **Total SNPs used for MR** |
| --- | --- | --- | --- |
| Type 1 diabetes | 59.64 | 0 | 50 |
| Type 2 diabetes | 43.82 | 0 | 227 |
| Hemoglobin A1C | 41.79 | 2 | 109 |
| Fasting Glucose - BMI adjusted | 37.55 | 3 | 91 |
| Fasting Insulin - BMI adjusted | 32.11 | 2 | 61 |
| Body Mass Index | 40.79 | 0 | 72 |
| C-reactive Protein | 44.12 | 0 | 446 |
| Waist-hip Ratio - BMI adjusted | 45.62 | 0 | 43 |
| Low Density Lipoprotein | 56.73 | 0 | 53 |
| High Density Lipoprotein | 54 | 1 | 63 |
| Triglycerides | 49.48 | 0 | 38 |
| Systolic Blood Pressure | 48.14 | 0 | 181 |
| Diastolic Blood Pressure | 44.64 | 0 | 183 |
| Creatinine-based eGFR | 42.68 | 0 | 280 |
| Chronic Kidney Disease | 45.77 | 0 | 21 |
| Coronary Artery Disease | 41.28 | 0 | 50 |
| Any Stroke | 27.97 | 0 | 16 |

MR: Mendelian randomization; BMI: Body mass index

**Table B.** Power calculations for Mendelian randomization analyses of COVID-19 positive and Hospitalization with COVID-19

| COVID-19 positive  Cases: 14,134  Controls: 1,284,876  Total sample: 1,299,010 | | | | |
| --- | --- | --- | --- | --- |
| Variance explained (%) | 1 | 3 | 5 | 10 |
| OR per SD | 1.40 | 1.23 | 1.18 | 1.13 |
| Hospitalization with COVID-19  Cases: 6,406  Controls: 902,088  Total sample: 908,494 | | | | |
| Variance explained (%) | 1 | 3 | 5 | 10 |
| OR per SD | 1.51 | 1.30 | 1.23 | 1.19 |

Power>0.8, alpha = 0.05/17/2 = 0.0015. The power analysis was performed using the shiny app: [**https://shiny.cnsgenomics.com/mRnd/**](https://shiny.cnsgenomics.com/mRnd/)
